# Enhanced FGFR3 activity in postmitotic principal neurons during brain development results in cortical dysplasia and axonal tract abnormality

**DOI:** 10.1038/s41598-020-75537-0

**Published:** 2020-10-28

**Authors:** Jui-Yen Huang, Bruna Baumgarten Krebs, Marisha Lynn Miskus, May Lin Russell, Eamonn Patrick Duffy, Jason Michael Graf, Hui-Chen Lu

**Affiliations:** 1 Department of Psychological and Brain Sciences, the Linda and Jack Gill Center for Biomolecular Sciences, Indiana University, 1101 E. 10th Street, Bloomington, IN 47405 USA; 2 Department of Psychological and Brain Sciences, Indiana University, Bloomington, IN 47405 USA; 3 Program in Neuroscience, Indiana University, Bloomington, IN 47405 USA

**Keywords:** Developmental biology, Neuroscience

## Abstract

Abnormal levels of fibroblast growth factors (FGFs) and FGF receptors (FGFRs) have been detected in various neurological disorders. The potent impact of FGF-FGFR in multiple embryonic developmental processes makes it challenging to elucidate their roles in postmitotic neurons. Taking an alternative approach to examine the impact of aberrant FGFR function on glutamatergic neurons, we generated a FGFR gain-of-function (GOF) transgenic mouse, which expresses constitutively activated FGFR3 (FGFR3^K650E^) in postmitotic glutamatergic neurons. We found that GOF disrupts mitosis of radial-glia neural progenitors (RGCs), inside-out radial migration of post-mitotic glutamatergic neurons, and axonal tract projections. In particular, late-born CUX1-positive neurons are widely dispersed throughout the GOF cortex. Such a cortical migration deficit is likely caused, at least in part, by a significant reduction of the radial processes projecting from RGCs. RNA-sequencing analysis of the GOF embryonic cortex reveals significant alterations in several pathways involved in cell cycle regulation and axonal pathfinding. Collectively, our data suggest that FGFR3 GOF in postmitotic neurons not only alters axonal growth of postmitotic neurons but also impairs RGC neurogenesis and radial glia processes.

## Introduction

The fibroblast growth factors (FGFs)-FGF receptors (FGFRs) family is comprised of structurally related tyrosine kinases receptors (FGFR1-4) and 22 FGF ligands^1,2^. These receptors interact with a large group of FGF ligands and initiate several different intracellular signaling pathways to regulate diverse biological processes^2,3^. Several FGFs/FGFRs are abundantly expressed in neuroprogenitors during early brain development^2,4–7^. Compelling studies demonstrate that FGFs-FGFRs are critical regulators of neurogenesis, neural differentiation, and cortical patterning in embryonic development^2,7–11^. During early mouse embryonic development, neocortical neurons are generated from embryonic day 10.5 (E10.5) when neuroepithelial progenitors begin to divide symmetrically to expand their numbers and differentiate into the radial glial cell (RGCs) that initiate neurogenesis^12,13^. It has been demonstrated that *Fgf10* promotes the transition of neuroepithelial cells to become RGCs^11^. Additionally, RGCs also act as stem cells that divide in an increasingly asymmetric manner to self-renew and generate restricted intermediate progenitor cells (IPCs) and neurons^14^. In this step, FGFR signaling in RGCs inhibits the transition from RGCs to IPCs and maintains the self-renewal ability of RGCs^4,15^. Taking into consideration the important function of FGF-FGFR signaling in governing neurogenesis, diminishing FGF-FGFR signaling results in loss of cortical surface area and a reduced number of glutamatergic neurons^5,15–18^. Conversely, a constitutively active FGFR3 mutation results in increased neuronal number and larger brain size^19,20^. In the late stage of cortical development (around E17.5 in mice), RGCs switch from the neurogenic phase to a gliogenic phase^21–23^; a recent study further demonstrates that FGF-FGFR signaling is necessary and sufficient to redirect cell fate from neurons to astrocytes^24^.

During early postnatal brain development, neuronal FGFRs are required to mediate neural activity-dependent processes in shaping dendritic patterning of cortical glutamatergic neurons^25,26^. FGF7 and FGF22 differentially regulate inhibitory and excitatory synapse formation in the hippocampus^27,28^. Taken together, FGFRs in postmitotic neurons are required for dendritic patterning and synaptogenesis. However, the role of FGF-FGFR signaling in cell-type specific differentiation of newborn neurons has yet to be explored. Here we employed the NEX-Cre line^29^ to express *FGFR3*^*K650E*^ (a constitutively active FGFR3) mainly in postmitotic glutamatergic neurons to elucidate the role of FGFR3 in neuronal differentiation and neural circuit wiring. Results from this gain-of-function (GOF) approach reveal the potent impacts of FGFR3 hyperfunction on cortical and hippocampal lamination, brain size, neuronal differentiation, and axonal pathfinding. Surprisingly, despite GOF of FGFR3 in *postmitotic neurons*, the proliferative capacity and radial processes of RGCs are impaired and result in defective cortical lamination.

## Results

### Expressing FGFR3^K650E^ in NEX-lineage postmitotic neurons results in aberrant cortical lamination

**Figure 1:**
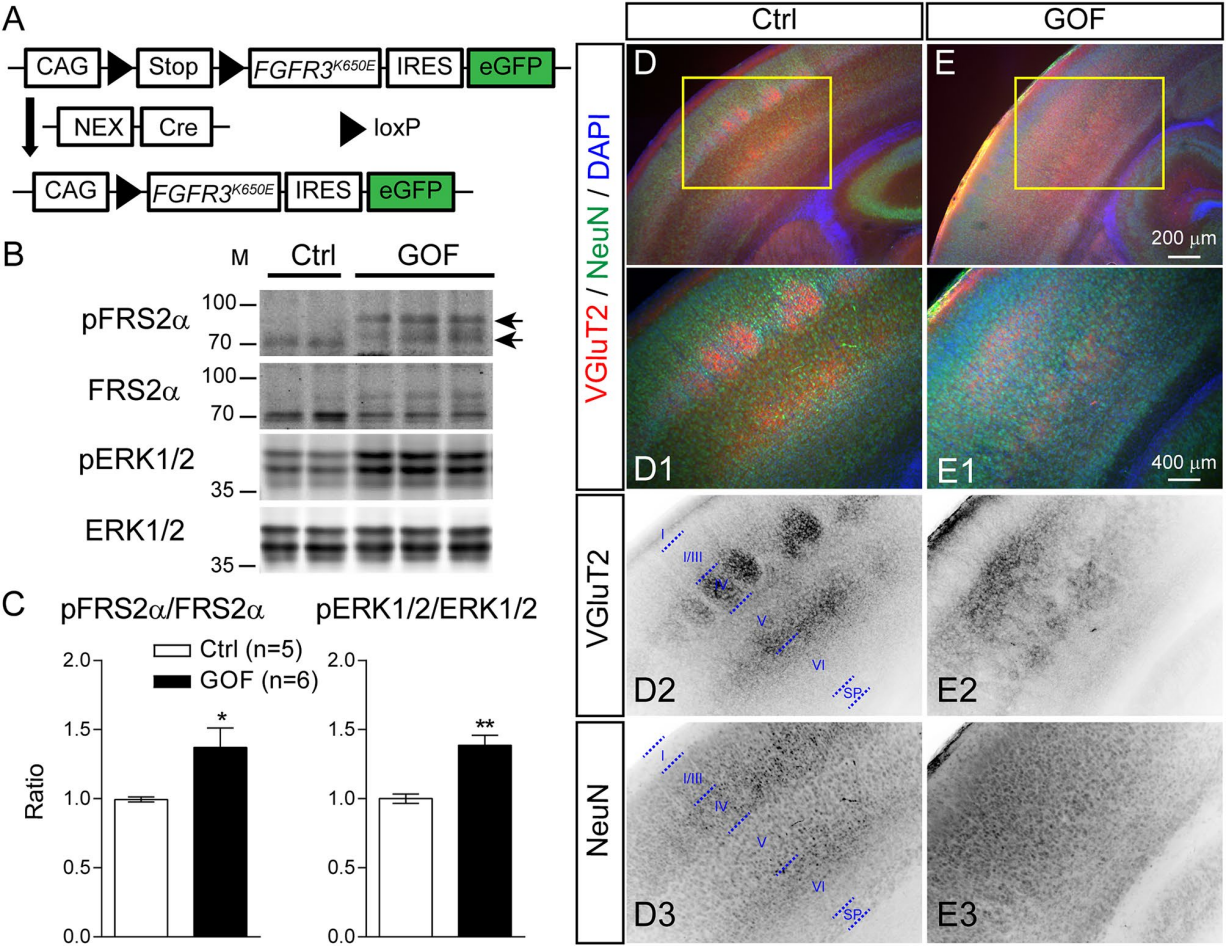
Expressing FGFR3^K650E^ in NEX-lineage neurons results in aberrant cortical lamination. (**A**) The diagram shows how GOF mice were generated. The black triangle indicates the loxP site. (**B**) Western blots show the abundance of phosphorylated FGFR substrate 2 α (FRS2α-Tyr196; pFRS2α), FRS2α, phosphorylated ERK1/2 (Thr202/Tyr204; pERK1/2), ERK1/2 in the S1 cortex of P7 control (Ctrl) and GOF mice. (**C**) Summaries for the fold changes in pFRS2α to FRS2α and pERK1/2 to ERK1/2 (Ctrl, n = 5; GOF, n = 6) in GOF mice. (**D**,**E**) VGluT2, NeuN, and DAPI staining in the P7 S1 cortex. D1-3 and E1-3 show the enlarged images in (**D**,**E**) (yellow boxes). D2-3 and E2-3 show the inverted images of individual channels. I–VI, cortical layers; SP, subplate. Student’s-t test. *, p < 0.05; ***, p < 0.001. See Supplementary Fig. 10 for full-length blots.

To explore the role of FGF-FGFR signaling in cortical neurons after neurogenesis, we expressed a constitutively activated FGFR3 mutant, *FGFR3*^*K650E*^, in postmitotic glutamatergic neurons. Specifically, we generated a FGFR gain-of-function (GOF) mouse model by crossing the transgenic mouse line carrying a *FGFR3*^*K650E*^ conditional allele, CAG-flox-stop-flox-*FGFR3*^*K650E*^-IRES-eGFP^30^ with the NEX-Cre mouse line^29^. The progenies carrying both NEX-Cre and *FGFR3*^*K650E*^ conditional alleles (abbreviated as GOF mice) express FGFR3^K650E^ in cortical and hippocampal glutamatergic neurons (Fig. 1A). FGFR activation can result in the phosphorylation on FGFR substrate 2 α (FRS2α) and extracellular signal-regulated kinase 1/2 (ERK1/2)^2^. Western blotting of cortices prepared from postnatal day 7th (P7) control and GOF mice reveals a significant increase in the abundance of phosphorylated FRS2α (p = 0.0402) and ERK1/2 (p = 0.0015), normalized to total FRS2α and ERK1/2, in GOF cortex compared to their littermate controls, verifying an increase of FGFR3 signaling (Fig. 1B,C). NEX-Cre mouse line expresses Cre in the postmitotic cortical neurons from E12^29^. Immunostaining study found significant increases of phosphorylated ERK1/2 signals in the cortical plate of GOF embryos at embryonic day 15.5 (E15.5 (Supplementary Fig. 1). Taken together, our data show that FGFR3 activity in GOF is enhanced in the cortical plate from embryonic stages.

The mouse primary somatosensory (S1) cortex is known for its distinctive whisker-related patterns, called barrels. Barrel formation requires coordinated development between the innervating thalamocortical axons and the migrating layer IV neurons. Barrel pattern can be disrupted by defective neuronal differentiation, neuronal migration, and axonal pathfinding^23,31,32^. To visualize the whisker-related patterns, we conducted immunostaining by using antibodies against NeuN (neuronal marker) and vesicular glutamate transporter 2 (VGluT2, thalamocortical axons marker)^33–35^ in P7 GOF and littermate controls. In control brains, VGluT2 signals were evident as whisker-related patches in cortical layer IV and a distinctive band in layer VI (Fig. 1D,D1,D2). The presence of VGluT2 verified that thalamocortical axons reached the S1 cortex of GOF mice. However, the distribution of VGluT2 signals in the GOF S1 cortex was dispersed throughout the middle of the cortical plate and lacked a distinctive pattern (Fig. 1E,E1,E2). Additionally, different from the lamination pattern revealed by NeuN in the control S1 cortex (Fig. 1D3), the distribution of NeuN in the GOF S1 cortex was rather uniform (Fig. 1E3).

Different from the typical tear-drop shape seen in control hippocampi (Supplementary Fig. 2A), GOF hippocampi were more rounded in shape (Supplementary Fig. 2B). Instead of the compacted pyramidal layers seen in control hippocampi (Supplementary Fig. 2C,C1), NeuN^+^ cells in GOF hippocampi were rather dispersed (Supplementary Fig. 2D,D1). Additionally, an ectopic cluster of neurons was often found directly above the CA1 region of GOF hippocampi (Supplementary Fig. 2B,D,D1). The neurons in the hilus of the dentate gyrus area were also dispersed more widely in GOF hippocampi (Supplementary Fig. 2F,F1), compared with control (Supplementary Fig. 2E,E1). The thickness of both the CA1 stratum pyramidale (p = 0.0014) and the hilus (p < 0.0001) were significantly greater in GOF compared to control mice (Supplementary Fig. 2G,H). Taken together, FGFR3 hyperfunction in glutamatergic neurons perturbs the lamination in the cortex and hippocampus.

### Postnatal FGFR3 GOF did not perturb cortical laminations

To determine the time window when FGFR3 GOF impact cortical lamination, we generated postnatal GOF mice by crossing the mice carrying CAG-flox-stop-flox-*FGFR3*^*K650E*^-IRES-eGFP allele with NEX-CreERT2 mice and activated Cre by injecting tamoxifen after birth (Supplementary Fig. 3A; abbreviated as postnatal GOF mice). Specifically, these time-specific GOF pups and littermate controls were subjected to 100 mg/kg tamoxifen via single intraperitoneal injection at postnatal day 1 (P1). The significant increases in the abundance of phosphorylated FRS2α (p = 0.0136) and ERK1/2 (p = 0.0373) in the P7 cortex from postnatal GOF pups (Supplementary Fig. 3B,C) confirmed the Cre-mediated recombination and upregulation of *FGFR3*^*K650E*^ in postnatal GOF pups. Immunofluorescent staining showed that GFP was expressed in the cortex and hippocampus of P7 postnatal GOF mice (Supplementary Fig. 3E,E1), but not control animals (Supplementary Fig. 3D,D1), again confirming appropriate Cre activation.

CUX1 is a homeodomain-containing DNA binding protein that is abundantly expressed in late-born cortical glutamatergic neurons^36,37^. In both control (Supplementary Fig. 3D,D2) and postnatal GOF mice (Supplementary Fig. 3E,E2), CUX1^+^ neurons were distributed throughout layer II-IV and are enriched in layer IV barrels. DAPI staining also revealed distinctive lamination and barrels in both control and postnatal GOF mice (Supplementary Fig. 3D3,E3). Taking together the embryonic GOF data, we found that FGFR3 hyperfunction in postmitotic glutamatergic neurons perturbed cortical lamination during late embryogenesis (between E12 and birth) but not after birth.

### Expressing FGFR3^K650E^ in NEX-lineage principal neurons resulted in misplacement of late-born neurons

**Figure 2:**
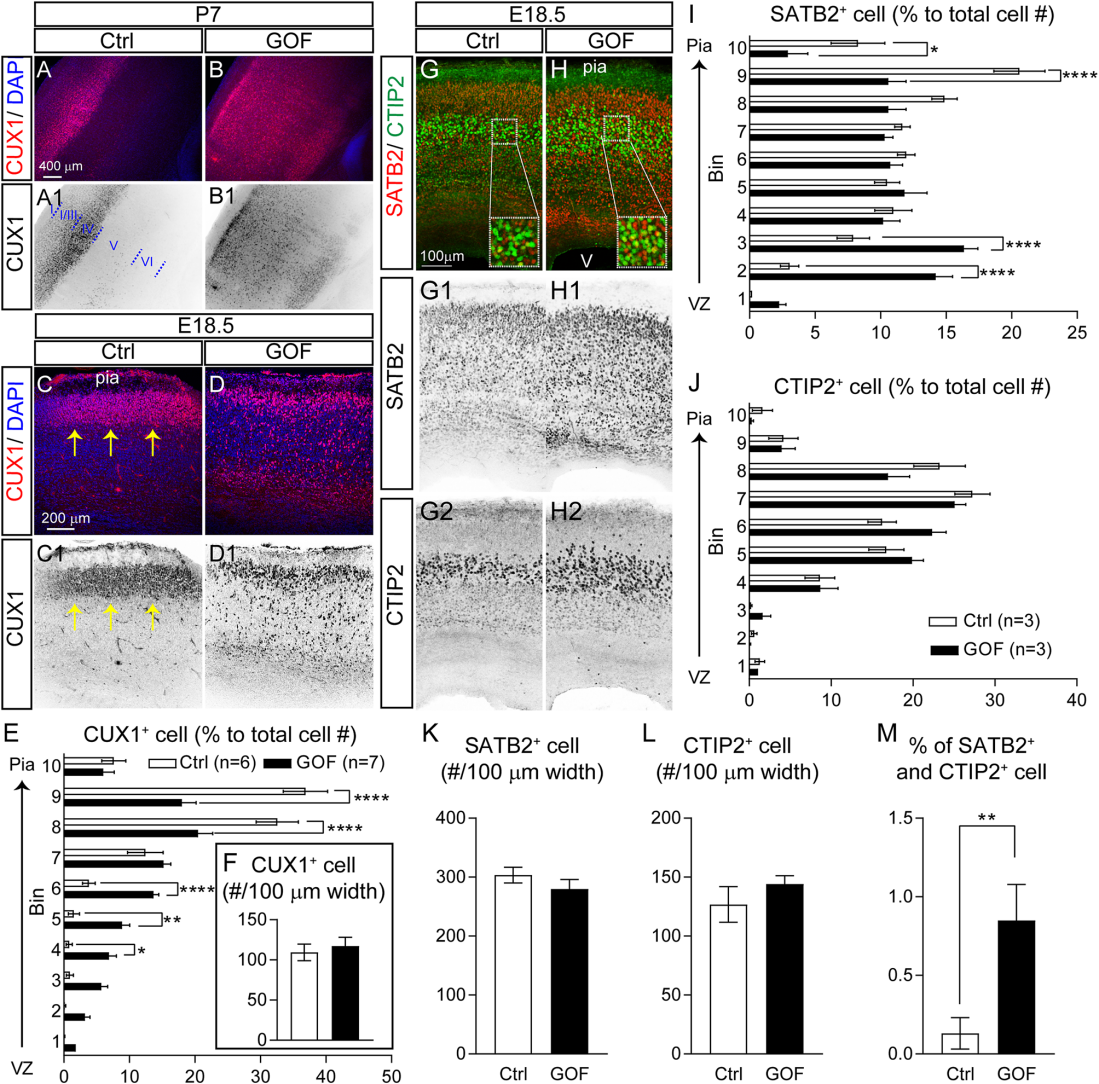
FGFR GOF in postmitotic neurons results in the misplacement of late-born principal neurons. (**A**,**B**) Example images of CUX1 staining with coronal sections prepared from the P7 control (Ctrl) and GOF S1 cortex. (**C**,**D**) CUX1 staining in E18.5 Ctrl and GOF brains. A1–D1 shows the inverted CUX1 staining images. The yellow arrows indicate the cluster of CUX1^+^ cells. (**E**) The distribution of CUX1^+^ cells in E18.5 cortex along different cortical depth. Two-way ANOVA post hoc Bonferroni’s multiple-comparisons test. The statistical analysis (*) compared between Ctrl and GOF for corresponding bins. *p < 0.05; **p < 0.01; ****p < 0.0001. (**F**) Summary of the total number of CUX1^+^ cells. Student’s *t*-test. (**G**,**H**) Example images for SATB2 and CTIP2 double staining with E18.5 Ctrl and GOF brains. G1-2 and H1-2 show the inverted images of SATB2 and CTIP2 staining. (**I**,**J**) Summaries for the distributions of SATB2^+^ and CTIP2^+^ cells alone the cortical depth in the E18.5 cortex. Two-way ANOVA post hoc Bonferroni’s multiple-comparisons test. (**K**,**L**) Summary graphs for the total numbers of SATB2^+^ and CTIP2^+^ cells. (**M**) Summary for the percentage of neurons co-expressing SATB2 and CTIP2. Student’s *t*-test. ** p < 0.01. *Pia* pial surface, *V* ventricle. All the images shown were in the coronal plane.

Cortical neurons are born in the ventricular zone and then migrate into the cortical plate to form cortical lamination in an inside-out manner with early-born neurons in the deep layer and late-born neurons in the upper layer of the cortical plate^23,38^. Lamination abnormalities often result from radial migration deficits^39–41^. To further explore if the lamination abnormality caused by FGFR3 GOF from the embryonic stage is due to misplacement of specific neuronal subtypes, we performed immunostaining with antibodies against cortical layer-specific markers CUX1, special AT-Rich Sequence-Binding protein 2 (SATB2), and COUP-TF-interacting protein 2 (CTIP2)^36,37,42^. Different from their upper layer localization found in control (Fig. 2A,A1), late-born CUX1^+^ cells were dispersed throughout P7 GOF cortical plate (Fig. 2B,B1). Such misplacement of CUX1^+^ neurons in the GOF cortex was also evident by E18.5 (Fig. 2C,C1,D,D1). In the GOF cortex, there were significantly fewer CUX1^+^ cells in the cortical plate (bin 8, p < 0.0001; bin 9, p < 0.0001), while more were present in the intermediate zone (IZ) and subventricular zones (SVZ) (bin 4, p = 0.0415; bin 5, p = 0.0057; bin 6, p < 0.0001) (Fig. 2E). There was no difference in the total number of CUX1^+^ cells between control and GOF mice (p = 0.6122; Fig. 2F). To further examine the distributions of different types of cortical neurons, SATB2 and CTIP2 double immunostaining was conducted with E18.5 brains (Fig. 2G,G1,2,H,H1-2). We found significantly more SATB2^+^ cells in the GOF brain distributed in deep layers (bin 2, p < 0.0001; bin 3, p < 0.0001), towards the ventricular zone (VZ) (Fig. 2I). CTIP2^+^ marks early-born glutamatergic neurons, and they are often found in the deep layer^36^. Distinct from CUX1^+^ or SATB2^+^ cells, the distribution of CTIP2^+^ cells is normal in the GOF cortex (Fig. 2J). The numbers of SATB2^+^ (p = 0.2685; Fig. 2K) and CTIP2^+^ (p = 0.3486; Fig. 2L) cells are similar between GOF and control mice. However, the percentage of SATB2 and CTIP2 double-positive cells is significantly higher in the GOF cortex (p = 0.005; Fig. 2M). To further examine if FGFR3 GOF has an impact on the migration of early-born neurons, we performed immunostaining with antibodies against T-box brain transcription factor1 (TBR1)^43,44^. We found that the majority of TBR1^+^ cells are distributed in the lower part of the cortical plate (bins 4–7) in both control and GOF brains (Supplementary Fig. 4). However, there is a slight but significant shift of TBR1^+^ cells in GOF brains towards the pial surface. Taken together, these results suggest that FGFR3 GOF in principal neurons disrupts the migration of postmitotic neurons, particularly affecting late-born cortical neurons.

### FGFR3 GOF in postmitotic principal neurons results in altered RGCs by reducing their mitosis potential and by losing the radial processes

**Figure 3:**
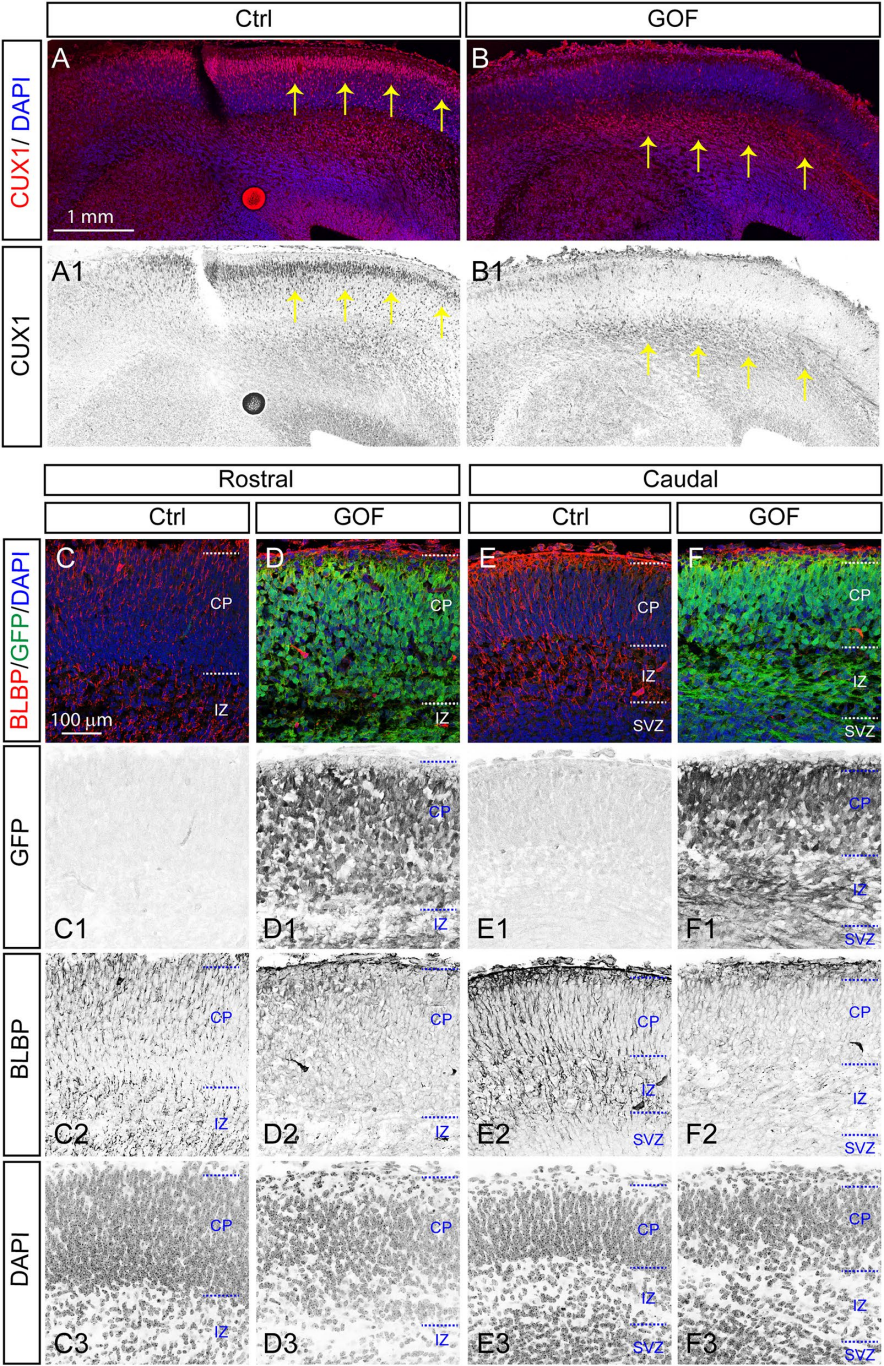
FGFR GOF in postmitotic principal neurons results in a loss of radial processes. (**A**,**B**) CUX1 staining with coronal sections prepared from E15.5 control (Ctrl) and GOF cortex. A1 and B1 show the inverted CUX1 staining images. Yellow arrows indicate the CUX1^+^ cell. (**C**–**F**) Images show BLBP and GFP staining in rostral (**C**,**D**) and caudal (**E**,**F**) plane at E15.5 embryos. C1-F1, C2-F2, and C3-F3 show the inverted GFP, BLBP, and DAPI staining images. The GFP^+^ cells are in both cortical plate and intermediate zone in GOF embryos. GFP expression in GOF embryos indicates the *FGFR3*^*K650E*^ allele is successfully activated at E15.5. Notably, BLBP^+^ processes are barely detected in the cortical plate and intermediate zone in GOF embryos. *CP* cortical plate, *IZ* intermediate zone, *SVZ* subventricular zone.

Newly born glutamatergic neurons radially migrate towards the pial surface via radial glia assisted radial migration^41,45^. The mislocalization of layer II–IV cortical neurons in GOF brains prompted us to examine the distribution of CUX1^+^ neurons and radial glia processes at E15.5 when radial migration is active. Radial glia processes are derived from PAX6^+^ RGCs and can be visualized by staining with an antibody against a brain-specific member of the lipid-binding protein (BLBP)^46^. We found that some CUX1^+^ cells already migrated to the upper part of the cortical plate at E15.5 (Fig. 3A,A1) while the majority of CUX1^+^ cells in the GOF brains remained at the bottom of the cortical plate (Fig. 3B,B1). We also found abundant BLBP^+^ radial processes in both rostral and caudal areas of the control embryonic cortex (Fig. 3C,C2,E,E2). However, BLBP^+^ radial processes were barely detectable in GOF embryos (Fig. 3D2,F2). The presence of GFP in the cortical plate of the GOF embryos indicated that Cre-mediated recombination occurred in postmitotic neurons located in the cortical plate region (Fig. 3D,D1,F,F1). There was no GFP expression in control embryonic brain tissue (Fig. 3C,C1,E,E1). Neurons in the cortical plate tended to align in a columnar fashion as a result of radial migration (Fig. 3C3,E3). In contrast, the arrangement of neurons in the GOF cortical plate appears rather disorganized (Fig. 3D3,F3). Results suggest that RGCs are affected by FGFR3 GOF in postmitotic neurons.

**Figure 4:**
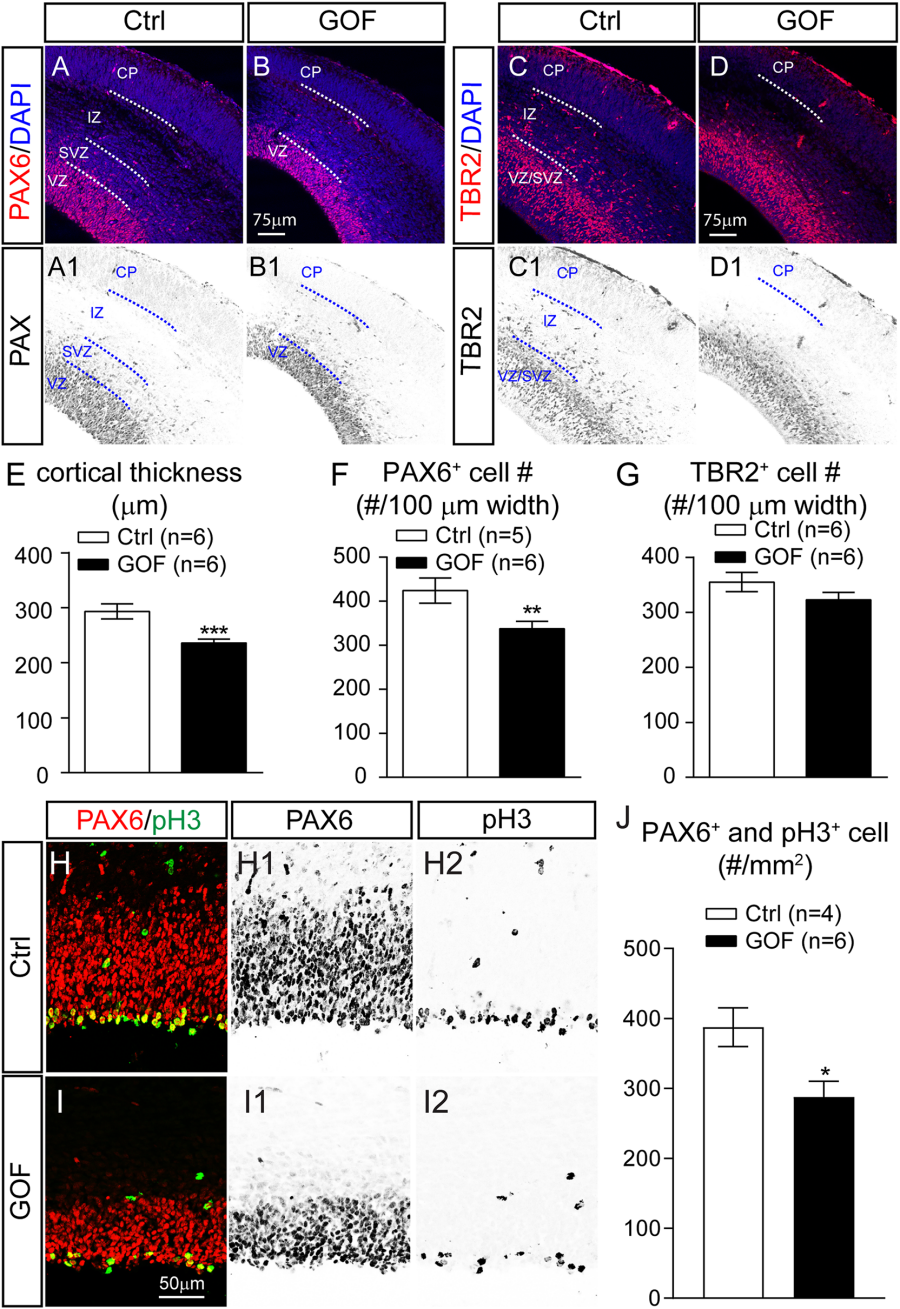
FGFR GOF in postmitotic principal neurons results in a reduction of RGCs in E15.5 brain. (**A**,**B**) PAX6 and DAPI staining with coronal sections prepared from control (Ctrl) and GOF cortex. A1 and B1 show the inverted images of PAX6 staining. (**C**,**D**) TBR2 and DAPI staining with coronal sections prepared from Ctrl and GOF cortex. C1 and D1 show the inverted images of TBR2 staining. *CP* cortical plate, *IZ* intermediate zone, *SVZ* subventricular zone, *VZ* ventricular zone. Summary of the measured cortical thickness (**E**), PAX6^+^ cell number (**F**), and TBR2^+^ cell number (**G**). (**H**,**I**) PAX6 and phospho-Histone H3 (pH3) double staining with coronal sections prepared from Ctrl and GOF cortex. H1 and I1 show the inverted images of Pax6 staining. H2 and I2 show the inverted images of pH3 staining. (**J**) Summary of the number of cells co-expressing PAX6 and pH3. Student’s *t*-test. *p < 0.05; **p < 0.01; ***p < 0.001.

RGCs not only assist newborn neurons to migrate in the appropriate direction, but they also act as stem cells that divide in an increasingly asymmetric manner to self-renew and generate restricted intermediate progenitor cells and neurons^12^. Thus, the finding that neuronal FGFR3 GOF impaired RGC process formation suggested that the function of neural precursor cells may also be compromised. To examine whether neuroprogenitor cells, PAX6^+^ RGCs, and TBR2^+^ IPCs^12–14,46–48^, were altered, we examined the abundance and distribution of PAX6^+^ and TBR2^+^ cells in the E15.5 cortex. In both control and GOF brains, PAX6^+^ staining revealed RGCs arranged as a packed layer at the base of the VZ (Fig. 4A,B), while TBR2^+^ immunoreactivity was mainly found in the SVZ/VZ (Fig. 4C,D). The thickness of the GOF cortex was significantly less than control (p = 0.0002; Fig. 4E). Quantification of GOF cortex revealed a significant reduction of PAX6^+^ RGCs (p = 0.0099; Fig. 4F), while the number of TBR2^+^ IPCs was normal in GOF cortex (p = 0.1571; Fig. 4G). However, there was no significant difference in the density of PAX6^+^ (Ctrl, 15,254 ± 1479 number/mm^2^ vs. GOF, 14,747 ± 993.4 number/mm^2^ ; p = 0.77) or TBR2^+^ (Ctrl, 12,181 ± 484.5 number/mm^2^ vs. GOF, 13,861 ± 789.2 number/mm^2^ ; p = 0.0834) cells, likely due to the reduced cortical thickness of GOF brains.

The decreased Pax6^+^ cells and reduced cortical thickness prompted us to examine the proliferation capacity of NPCs. The NPCs engaged in S-phase were labeled by a 2-h IdU pulse at E15.5 and quantified (Supplementary Fig. 5). We found no difference in the distribution or total number of IdU^+^ cells in control and GOF embryonic cortices. Next, we examined the mitosis potential of PAX6 + cells with phospho-Histone H3 (pH3) staining, labeling cells in the G2-M transition and M phases of the cell cycle^49^ (Supplementary Fig. 5). The majority of pH3^+^ cells were located next to the ventricle in both control and GOF embryonic brains. However, the densities of pH3^+^ cells (Ctrl, 410.9 ± 29.26 number/mm^2^ vs. GOF, 310.4 ± 28.7 number/mm^2^; p = 0.0297) and PAX6-pH3 double-positive cells were significantly decreased (p = 0.0124) in GOF E15.5 brain (Fig. 4J).

To confirm that the loss of radial glia processes and reduced number of PAX6^+^ cells were due to non-cell autonomous influences of FGFR3 GOF from postmitotic neurons instead of leaky Cre expression in PAX6^+^ cells from NEX-Cre line, we examined if NEX-Cre can induce recombination in PAX6^+^ cells. E15.5 embryos carrying one copy of the tdTomato reporter gene (Ai9, Rosa-CAG-LSL (stop)-tdTomato) and one copy of the NEX-Cre allele were generated (Supplementary Fig. 6A) and collected for PAX6/TBR2 double immunostaining (Supplementary Fig. 6B). TdTomato^+^ cells, reporting Cre-mediated recombination, are mainly localized at the cortical plate (Supplementary Fig. 6C,H), and their axons projected through IZ (Supplementary Fig. 6C,E,G). No tdTomato^+^ cells were found to be PAX6^+^ or TBR2^+^ (Supplementary Fig. 6D,F). Taken together, our results suggest that FGFR3 GOF in postmitotic principal neurons imparts non-cell autonomous effects on RGCs.

### FGFR3 GOF in postmitotic principal neurons decreases the number of neurons and glial cells and brain size

**Figure 5:**
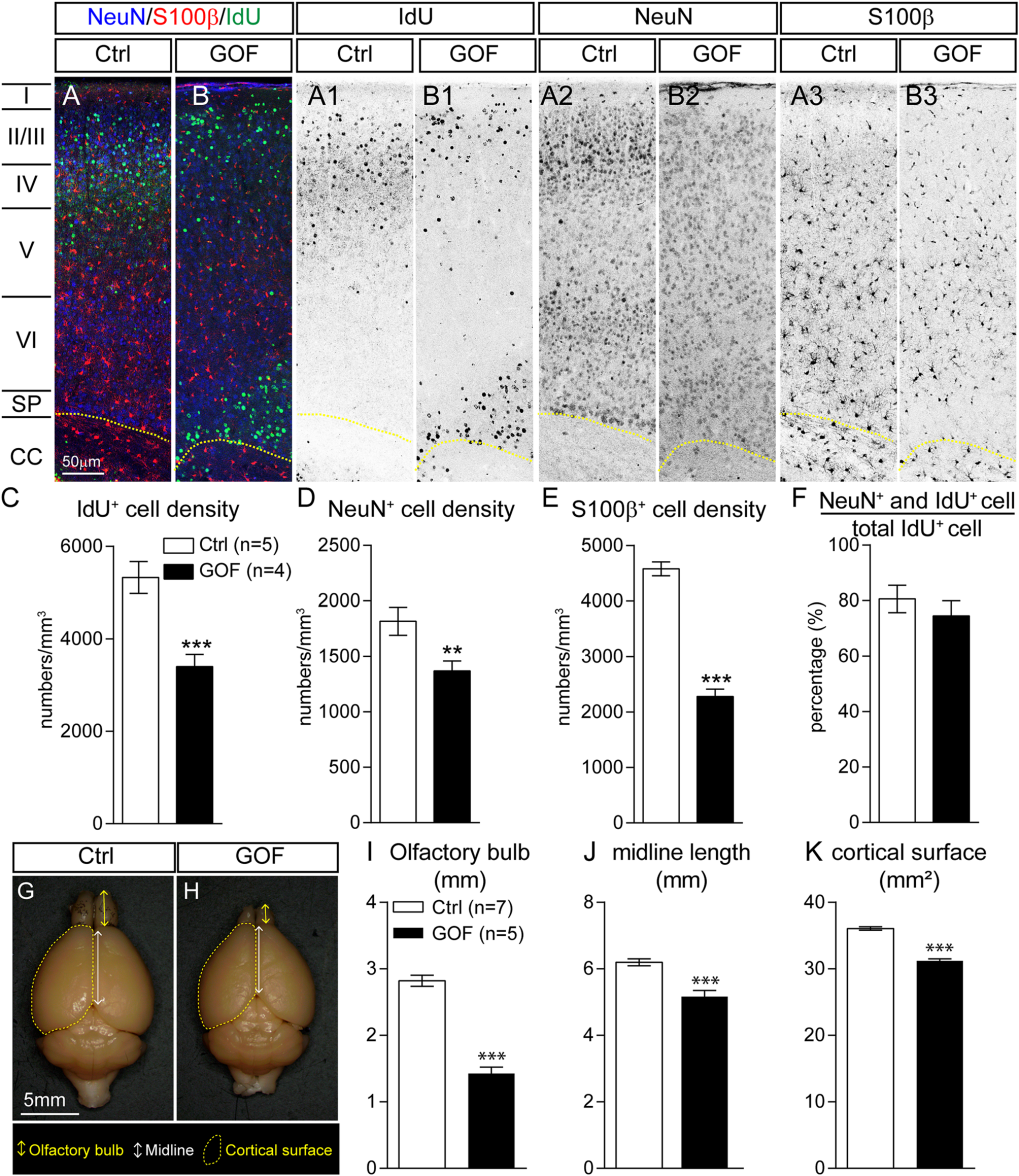
FGFR GOF in postmitotic principal neurons decreases neurons, glia, and brain size. Time-pregnant females at E14.5 were injected with IdU, and their progenies were examined at P7. (**A**,**B**) Representative images show NeuN, S100β, and IdU staining in P7 Ctrl and GOF S1 cortex. A1–A3 and B1-B3 show the inverted images of IdU, NeuN, and S100β staining. Summaries for IdU^+^ (**C**), NeuN^+^ (**D**), S100β^+^ (**E**) cell densities as well as the percentage of NeuN^+^-IdU^+^ double-positive cells to IdU^+^ cells (**F**). (**G**,**H**). Images (dorsal view) of brains harvested from P121 Ctrl and GOF mice. The olfactory bulb length (**I**), midline length (**J**), and cortical surface area (**K**) in G and H were measured (Ctrl, n = 7; GOF, n = 5) with photos in 2-D. Student’s *t*-test. **p < 0.01; ***p < 0.001; ****p < 0.0001. *I–VI* cortical layers, *SP* subplate, *CC* corpus callosum.

To determine whether the reduction of PAX6^+^ RGCs in the GOF embryonic cortex results in fewer cells in the postnatal brain, IdU was injected at E14.5 to label cells born at E14.5 and then the number of IdU^+^ cells in the S1 cortex was examined at P7 (Fig. 5A,B). We found that the number of IdU^+^ cells (p = 0.0001; Fig. 5A1,B1,C), NeuN^+^ cells (neurons, p = 0.0061; Fig. 5A2,B2,D), and S100β^+^ cells (astrocytes, p < 0.0001; Fig. 5A3,B3,E) were significantly decreased in the GOF cortex. However, there was no significant difference in the ratios of NeuN-IdU double-positive cells to total IdU^+^ cells between GOF and control cortex (p = 0.4165; Fig. 5F). We also evaluated brain size by imaging the dorsal side of fixed adult GOF and control brains (Fig. 5G,H) and measured the projected dimensions. We found that the 2D projected length of olfactory bulb (p < 0.0001; Fig. 5I) and midline (p = 0.0004; Fig. 5J) of fixed brains as well as the projected cortical surface area (p < 0.0001; Fig. 5K) were significantly less in the GOF mice compared to control. Our results suggest that FGFR3 GOF solely in postmitotic neurons results in fewer RGCs, decreases the number of neurons and astrocytes in the cortex, and decreases brain size.

### Unbiased transcriptomic analysis reveals putative cellular mechanisms on how FGFR GOF impacts RGCs and neuronal differentiation

**Figure 6:**
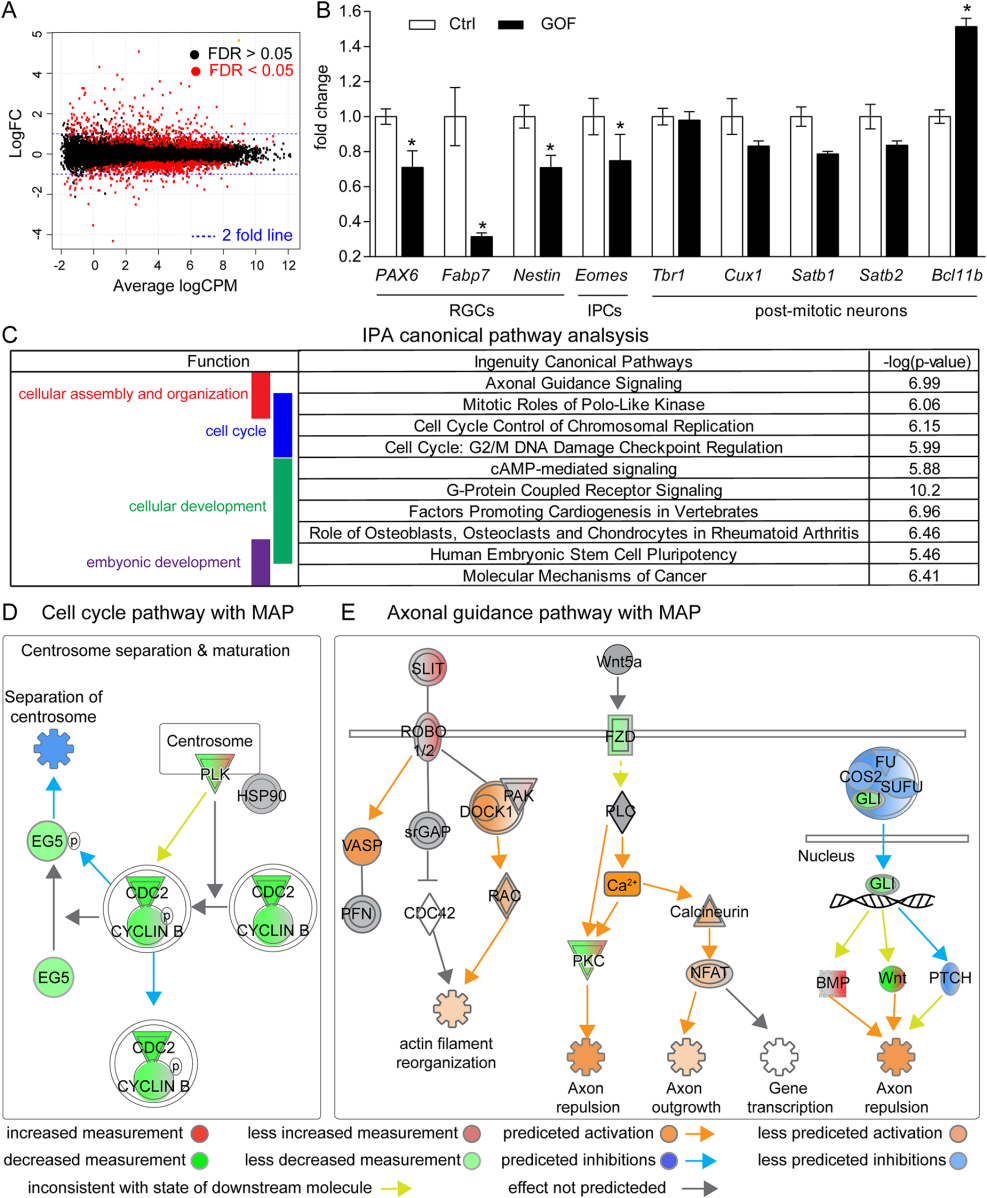
IPA analyses for differentially expressed genes found in E15.5 GOF embryonic cortex. (**A**) The smear plot demonstrates the log-fold change (FC) against average log counts per million reads (CPM). The black and red dots represent genes with FDR > 0.05 and FDR < 0.05, respectively. Blue lines indicate the twofold change lines. (**B**) Transcriptional changes of genes associated with RGCs (*Pax6*, *Fabp7, Nestin*), IPCs (*Eomes*), and postmitotic neurons (*Tbr1*, *Cux1*, *Satb1*, *Satb2*, *Bcl11b*) summarized from RNA-seq data. (**C**) Top 10 significant canonical pathways identified by IPA classified by physiological function. These pathways are involved in regulating cellular assembly and organization, cell cycle, cellular development, and embryonic development (shown in the left panel). (**D**,**E**) Schematic diagrams show the partial pathways related to cell cycle and axonal guidance with the molecule activation prediction (MAP) analysis. Molecules are indicated by standard abbreviations. Relative changes in gene expression are depicted by graduated shades of color coding: red, up; green, down; white, no change, or not applicable. MAP analysis in IPA predicts the activation status of each pathway component based on the transcriptional state of the relevant genes. Relative activation and inhibition status are depicted by graduated shades of color coding: orange, activation; blue, inhibition; yellow, inconsistent with the state of the downstream molecule; grey, effect not predicted.

To further explore the putative molecular mechanisms on how FGFR3 GOF impacted cortical development, RNA-sequencing (RNA-seq) was conducted with RNA prepared from E15.5 control and GOF embryonic cortices, as the mislocalization of CUX1^+^ cells was already evident at E15.5 (Fig. 3). RNA-seq studies found that FGFR3 GOF in glutamatergic neurons resulted in 537 genes upregulated and 596 genes downregulated (FDR < 0.05; Fig. 6A, Supplementary Dataset 1). Based on the RNA-seq data, mRNA levels of RGC markers such as *Pax6*, *Fabp7* (encoded brain-specific member of the lipid-binding protein, BLBP, which is located in RGC processes), and *Nestin* as well as the IPC specific gene marker *Enomes* (TBR2, IPCs) were decreased in GOF cortices (Fig. 6B). Additionally, mRNA levels of several neuronal markers such as *Tbr1*, *Cux1*, *Satb1*, *Satb2* did not differ between control and GOF cortices (Fig. 6B). An exception to this was that the expression of *Bcl11b* (encoded CTIP2), a marker of deep layer cortical neurons, was significantly upregulated (Fig. 6B).

We further conducted pathway analysis with Ingenuity Pathway Analysis (IPA; Qiagen) to aid in the interpretation of downstream biological pathways affected by FGFR3 GOF. Pathway analysis found 185 canonical pathways that had z-scores either considerably greater or considerably less than 0 and for which the up-down-regulation was statistically significant, including expected FGF signaling (Z score = 0.577) and ERK/MAPK signaling (Z sore = 0.943) (Supplementary Dataset 2). When the top 10 significant canonical pathways were broadly classified based on physiological function as described in the IPA database, pathways involved in regulating cellular assembly and organization, cell cycle, mobile development, and embryonic development were prominent (Fig. 6C).

The decrease of RGC number prompted us to explore further the cell cycle-regulated pathways in-depth. We found pathways that regulate the cell cycle were specifically related to mitosis most affected (Fig. 6D). To determine the activation state of the pathways identified from IPA analysis, we performed a molecule activation prediction (MAP) analysis in IPA. This bioinformatics tool predicted the activation states of each pathway component based on the transcriptional state of the relevant genes. MAP analysis showed that the centrosome separation pathway was inhibited (Fig. 6D). In the pathway of centrosome separation and maturation, several vital downstream effector proteins such as kinesin family member 11 (EG5), cell division control protein 2 (cdc2), and cyclin B were downregulated in FGFR3 GOF embryos (Fig. 6D). Moreover, we also found that a total of 50 genes related to axonal guidance signaling pathways were altered in FGFR3 GOF embryos including *Slit2*, *Slit3*, *Robo1, Fzd1, Fzd2, Fzd8, Gli3,* and *Wnt* (Fig. 6E). MAP analysis predicted that several events related to axonal outgrowth such as actin filament reorganization, axon repulsion, and axon outgrowth, are activated (Fig. 6E). Taken together, the transcriptomic profile suggests that FGFR3 GOF in postmitotic glutamatergic neurons exerts non-cell autonomous influences on cell cycle progression of NPCs’s and a cell-autonomous impact on neuronal pathfinding.

### FGFR3 GOF in postmitotic principal neurons results in severe axonal tract abnormalities

**Figure 7:**
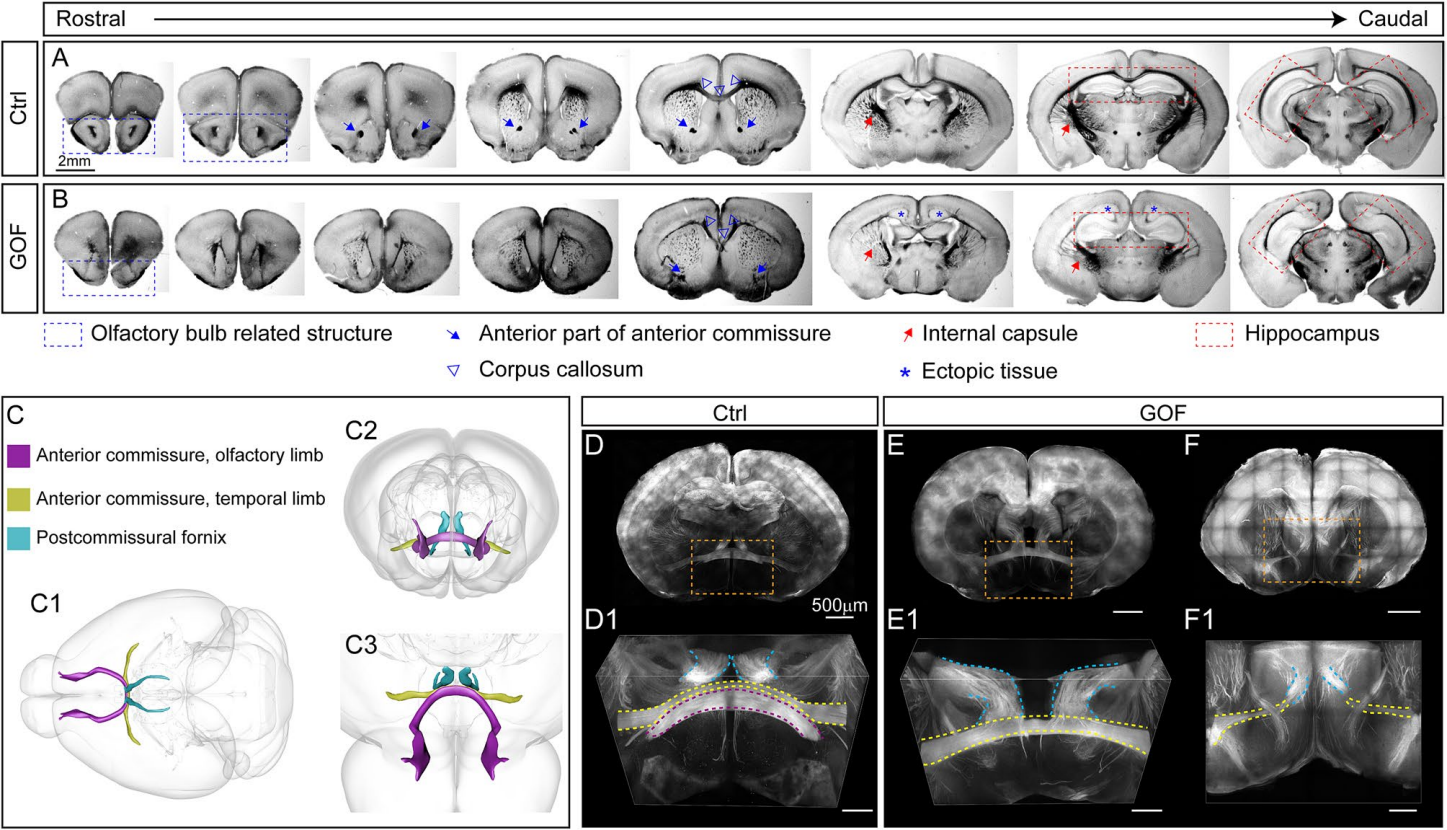
FGFR3 GOF mice have aberrant trajectories of anterior and posterior commissures. (**A**,**B**) Representative images show the anatomical features of coronal brain sections from rostral to caudal area in control (Ctrl) and GOF mice. The olfactory bulb related structures are marked with blue rectangles. The blue arrows indicate the anterior part of the anterior commissure. The red arrows indicate the internal capsule. The blue arrowheads indicate the corpus callosum. Blue stars mark the ectopic cell clusters in GOF mouse brains. The red rectangles mark hippocampal regions. (**C**) Scheme illustrating the axonal projections in 3-D. C1 shows the dorsal view. C2 shows the front view from the rostral side, which is like the images in D-F. C3 represents the viewing orientation as the images in D1-F1. (**D**–**F**) The fluorescence images from Ctrl and GOF coronal brain slices. D1, E1, and F1 show the enlarged views for the area indicated with orange rectangles in (**D**–**F**). The arch-shaped olfactory limb of the anterior commissure is highlighted with purple dashed lines. The temporal limb of the anterior commissure (labeled by dashed yellow lines) is located in front of the columns of postcommissural fornix (marked by dashed blue lines). *D* dorsal, *V* ventral, *A* anterior, *P* posterior.

FGF-FGFRs have been shown to regulate axonal pathfinding during embryonic development^50–53^; thus, we further examined if FGFR3 GOF perturbed axonal trajectories. Serial brain sections were prepared and imaged under a dissecting bright-field microscope (Fig. 7A,B). Several anatomical deficits in GOF mice were evident even at this gross level of evaluation. First, olfactory bulb related structures, including the anterior olfactory nucleus (AOD) and anterior olfactory nucleus external part (AOE), were smaller or not fully developed (Fig. 7B) in GOF mice. In control mice, the anterior part of the anterior commissure (ACA) passed through several coronal sections as a bundle passing through the ventral part of the brain (Fig. 7A). However, ACA fibers were not evident or were shifted more laterally in the GOF brain (Fig. 7B). The projection pattern of the internal capsule, which contains axonal projection fibers, including cortical thalamic, cortical striatal, and thalamocortical projections, was reduced and deformed in GOF mice (Fig. 7B). The thickness of the corpus callosum was greatly reduced (Fig. 7B). We also observe ectopic cell clusters in GOF mouse brains (Fig. 7B). The overall shape of the hippocampi was quite round in GOF mice (Fig. 7B).

The evident fiber tract abnormality at gross anatomical levels motivated us to examine further the impact of FGFR3 GOF on axonal trajectories that are originating from NEX-lineage neurons in 3 dimensions (3-D) in P7 brains. Thus, we generated the triple transgenic line with tdTomato reporter to label the axonal projections of NEX-lineage neurons with or without FGFR3 GOF. While the 2-D bright-field images reveal gross anatomical differences, examining brain tissue at the 3-D level provides a more comprehensive understanding of axonal trajectories. With the combination of the ScaleS tissue clearing methods^54^ and two-photon microscope imaging, we acquired image stacks of 1 mm-thick coronal brain sections to examine the axonal projection in 3-D (Fig. 7D–F, Supplementary Fig. 7). We first examined the most anterior 1 mm slices, which contain parts of the olfactory and temporal limbs of the anterior commissure as well as the postcommissural fornix (Fig. 7C). The olfactory limb of the anterior commissure showed an “arch shape” (Fig. 7D) in control mice, yet this structure is missing in GOF mice (Fig. 7E,F). The temporal limb of the anterior commissure, located in front of the postcommissural fornix, was observed in all control mice (Fig. 7D1, Supplementary Fig. 7) but only in some GOF mice (Fig. 7E1, Supplementary Fig. 7). Intriguingly, some GOF mice had both apparent aberrant temporal limb of the anterior commissure that were not crossing midline and abnormal trajectories (Fig. 7F1, Supplementary Fig. 7). In summary, the posterior portion of the olfactory limb of the anterior commissure appears to be heterogeneously altered in GOF mice.

**Figure 8:**
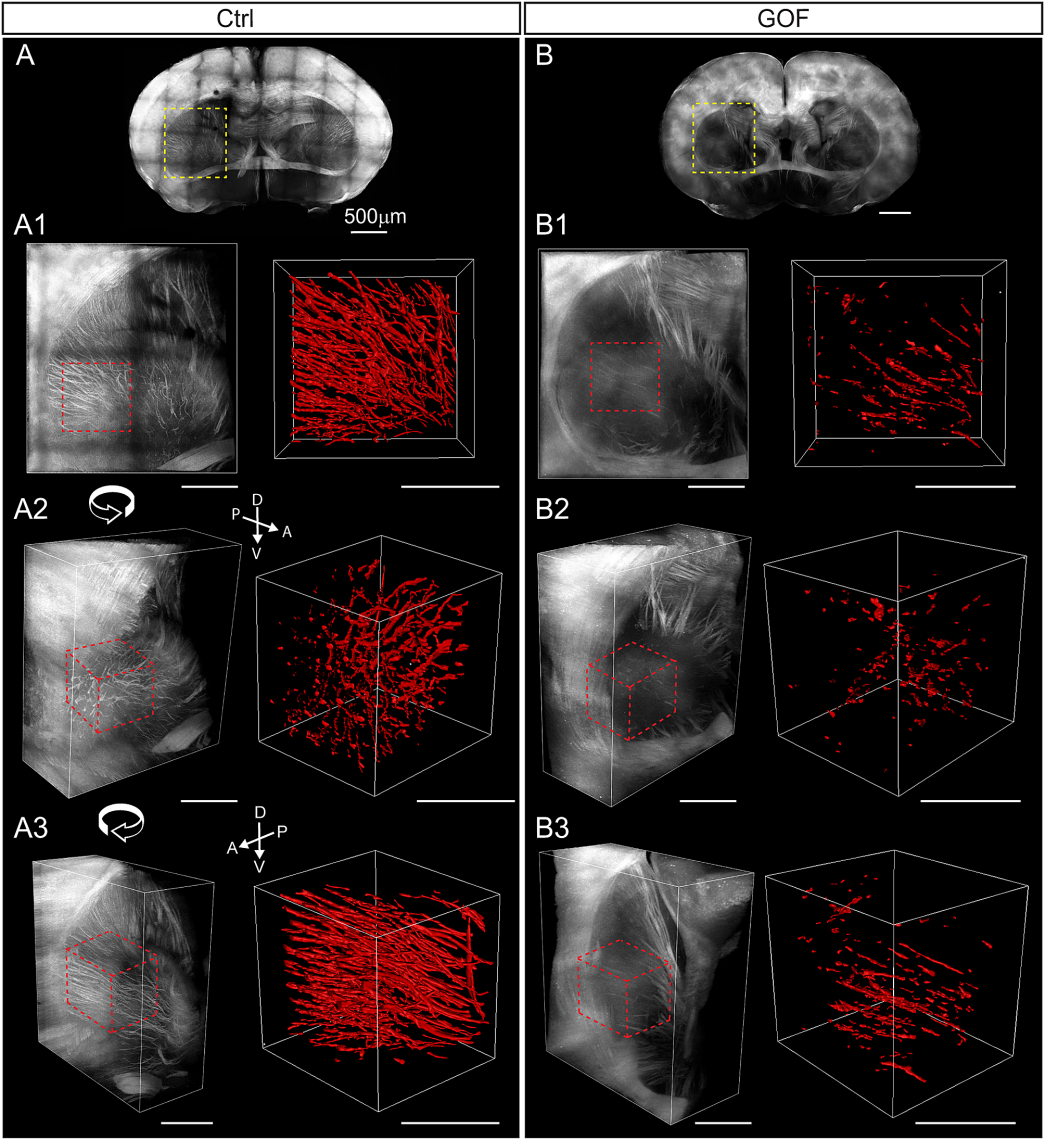
FGFR3 GOF mice have fewer axonal projections in the striatum. (**A**,**B**) Overview of coronal brain slices from control (Ctrl) and GOF mice. A1-A3 and B1-B3 show the zoomed-in view with different rotation angles in the striatum (yellow rectangle in **A**,**B**). The region analyzed in Imaris is marked with dashed red lines. The right panel in A1-A3 and B1-B3 shows the 3-D rendering of axonal fibers in the striatum (dotted-line delimited area in the left panel). *D* dorsal, *V* ventral, *A* anterior, *P* posterior.

TdTomato^+^ axonal projections reveal cortico-striatal and cortico-thalamic projections in the striatum in control mice (Fig. 8, Supplementary Fig. 8). We selected a portion of the striatum located in a similar position and applied Imaris surface rendering function to reconstruct the tdTomato^+^ axonal tracts in both control (Fig. 8A) and GOF brains (Fig. 8B). The quantification reveals that tdTomato^+^ axonal projections in the selected striatal area occupied a significantly smaller volume in GOF than control mice (Ctrl, n = 5, 17.7 ± 3.096 × 10^6^ µm^3^ vs. GOF, n = 4, 2.595 ± 0.5197 × 10^6^ µm^3^; p = 0.0037). Taken together, the number of NEX-lineage axonal projections present in the striatal area is significantly reduced in GOF mice.

**Figure 9:**
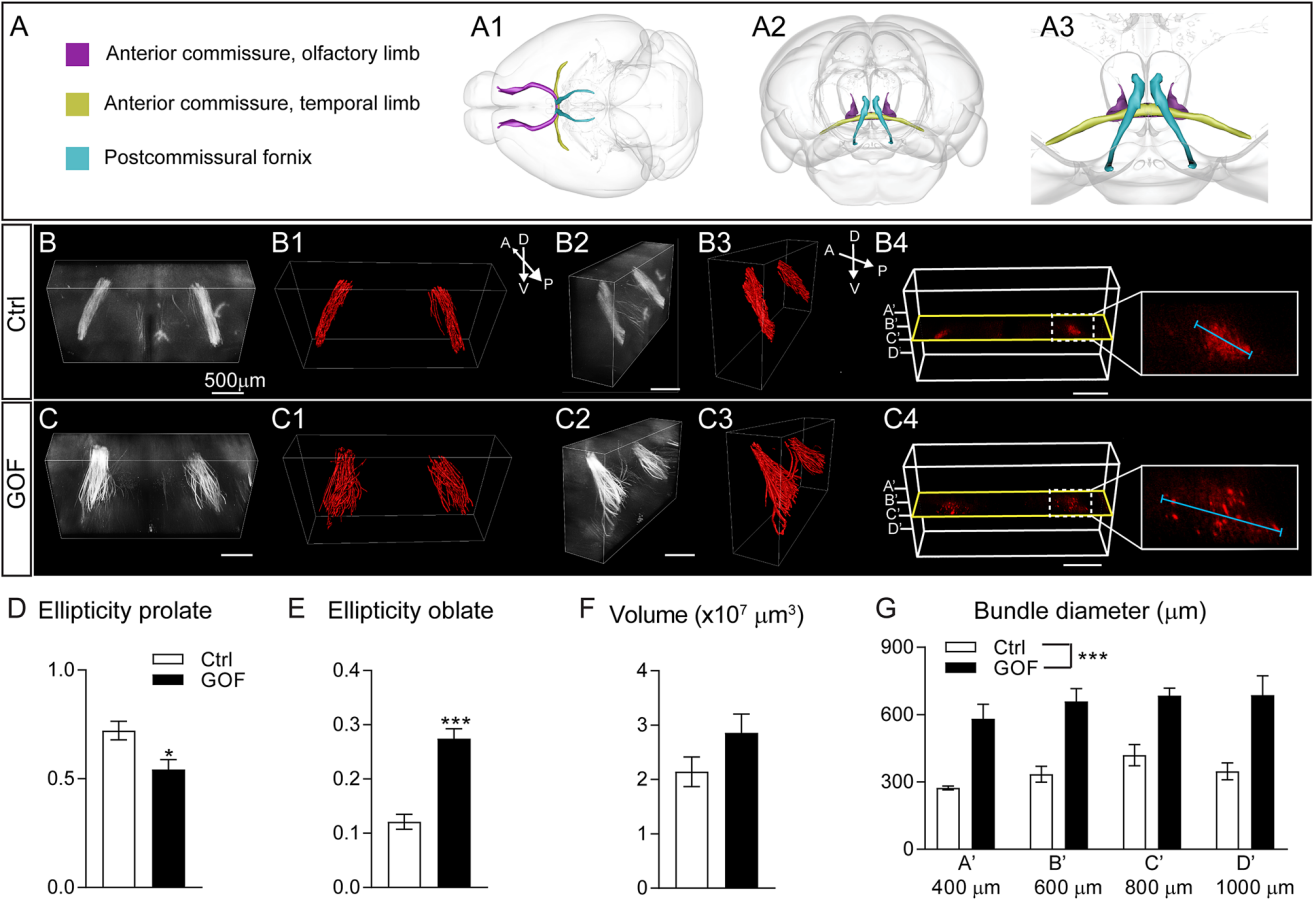
FGFR3 GOF disrupts axonal fasciculation of the postcommissural fornix. (**A**) Scheme illustrating the axonal projections in 3-D. A1 shows the dorsal view. A2 shows the front view from the rostral side. A3 represents the enlarged view in A2 (**B**,**C**) The front views from the rostral side of the postcommissural fornix bundles from Ctrl and GOF mice. B2 and C2 show the side view of (**B**,**C**). B1, B3, C1, and C3 show the Imaris 3-D surface rendering images. (B4, C4) Scheme illustrating the image planes used to measure the diameter of the postcommissural fornix bundle. 2-D images from four planes (A’, B’, C’, D’ at 400 µm, 600 µm, 800 µm, and 1000 µm) were extracted, and the longest diameter of the axonal bundle in each plane was measured. Summaries for prolate ellipticity (**D**), oblate ellipticity (**E**), total volume occupied (**F**) by postcommissural fornix projections. Student’s *t*-test. * p < 0.05; ***p < 0.001. (**G**) Summary of the diameters of postcommissural fornix bundles at four different planes indicated in B4 and C4. Two-way ANOVA post hoc Bonferroni’s multiple-comparisons test. *D* dorsal, *V* ventral, *A* anterior, *P* posterior.

The postcommissural fornix, the major output from the hippocampal subiculum, innervates the anterior hypothalamus through the medial-cortico hypothalamic tract and terminates at the posterior hypothalamus in the mammillary body^55,56^. We utilized the same approach described above to quantitatively characterize the postcommissural fornix in control and GOF brains (Fig. 9, Supplementary Fig. 9). The bundles of the postcommissural fornix in control brains are tightly packed and exhibit a prolate ellipsoid or “cigar-shape” (Fig. 9B1–B3) as illustrated in cartoon figures (Fig. 9A). However, these postcommissural fornix bundles in GOF brains are loosely packed and resemble an oblate ellipsoid, or a “disc-shaped” structure (Fig. 9C1–C3). These bundles in GOF mice have a significantly smaller value of prolate ellipticity (p = 0.0102; Fig. 9D) and a significantly higher value of oblate ellipticity (p < 0.0001; Fig. 9E) compared to control bundles. There is no significant difference in total volume occupied by tdTomato^+^ axonal projections (p = 0.1407, Fig. 9F) between the two lines. These data suggest similar numbers of axons between Ctrl and GOF postcommissural fornix. To further determine if the shape change is caused by axonal fasciculation deficits by FGFR3 GOF, we analyzed the diameter of the bundles in 4 horizontal planes extracted from the 3-D volume for both control and GOF brains (Fig. 9B4,C4). There was a significant increase in the diameter of the postcommissural fornix bundles found in GOF compared to control mice (p < 0.0001, Fig. 9G). Results indicate that FGFR3 GOF perturbs the axonal fasciculation of the postcommissural fornix.

## Discussion

In this study, we test specifically how FGFR3 GOF in the postmitotic neurons impact brain development. Using the GOF approach, we extend previous studies to reveal that FGFR signaling hyperactivation exerts significant adverse effects on cortical development. FGFR3 GOF in postmitotic neurons is sufficient to impart non-cell autonomous effect on RGCs, reducing RGCs numbers and function, and exert cell-autonomous effect to affect axonal guidance, resulting in overall abnormal cortical lamination and axonal tract abnormalities. The results reported here substantially extend our understanding of neuronal FGFR action in CNS.

### FGFR3 GOF in NEX-lineage neurons leads to aberrant cortical and hippocampal lamination

Radial migration guided by RGCs is a critical cellular process in cortical lamination^45,57,58^. Hippocampal neurons located in the CA1 region also require radial glial fibers for their appropriate migration^59^. The significantly reduced RGCs’ radial processes in the cortical plate of GOF embryonic brains (Fig. 3D,F) suggest that the defective radial migration can account for the lamination defects. It has been shown that early- and late-born neurons use different migratory strategies to arrive at their destinations^60,61^. Both early- and late-born neurons use somal translocation to reach the cortical plate. However, the late-born neurons farther migrate along the processes of radial glial cells to reach the top of the cortical plate. The observation that migration defects strongly affect late-born (CUX1^+^ and SATB2^+^) but much less on early-born (CTIP2^+^ and TBR1^+^) cortical neurons (Fig. 2 and Supplementary Fig. 4) suggests that GOF disrupt radial migration but less on somal translocation. For the early-born neurons, we found a slight but significant shift of Tbr1 distribution toward pial surface while remain clustered (Supplementary Fig. 4). Such changes can be caused by the reduced thickness of the cortical plate in GOF brains. Alternatively, this distribution difference might be caused by migration deficits of late-born cortical neurons. Many late-born neurons failed to migrate toward the pial surface and remain near the ventricular zone, subsequently altering the distribution of TBR1^+^ cells. Interestingly, deleting GSK3 using NEX-Cre resulted in radial migration and lamination phenotypes^62^, similar to the phenotypes we observed in GOF brains. Morgan-Smith et al*.* showed that GSK3 deletion delays the multipolar to bipolar transition of migrating cortical neurons. It has been demonstrated that FGFR signaling activates Akt signaling^63^, which subsequently inhibits GSK3 activity by phosphorylating GSK3. Thus, it is plausible that FGFR3^K650E^ expression in cortical neurons results in reduced GSK3 signaling and alters neuronal migration.

### FGFR3 GOF in NEX-lineage postmitotic neurons disrupts the cell-cycle progression of RGCs

The formation of neural circuits within the central nervous system depends upon the precise spatially and temporally coordinated generation of distinct classes of neurons and glia from RGCs and IPCs^23,64^. Our finding that PAX6^+^ RGCs are decreased in GOF embryonic cortex (Fig. 4) likely accounts for the reduced number of neurons and astrocytes in postnatal GOF brains as well as their smaller brain size (Fig. 5). IPA analysis of the transcriptomic profile of the E15.5 GOF embryonic cortex identifies that canonical pathways related to cell cycle progression are altered (Fig. 6). Specifically, MAP analysis predicted that the downregulation of polo-like kinase (PLK1) may be the upstream protein to suppress cell cycle progression (Fig. 6D, Supplementary Dataset 2). PLK1, a serine/threonine-protein kinase, has been recognized as a critical regulator of mitosis, meiosis, and cytokinesis^65,66^ and is expressed in VZ/SVZ^67^. It remains to be experimentally validated for the pathways identified by bioinformatics with future experiments and if FGFR3 GOF impairs the proliferation of RGCs.

The number of neurons in the postnatal brain is regulated by neurogenesis and cell death^68–73^. We found that GOF reduced the number and proliferative potential of PAX6^+^ RGCs, while the number of IPCs was normal. However, normal CUX1^+^, SATB2^+^, CTIP2^+^, and TBR1^+^ cell densities were observed in E18.5 GOF cortex. It is possible that the increased proliferative potential of Tbr2^+^ IPCs, reduced cell death, or both lead to the normal cell densities seen in E18.5 GOF cortex. Intriguingly, the density of NeuN^+^ neurons is significantly reduced in the P7 GOF cortex. Previous work found that the numbers of Nex-lineage neurons decrease during P2–P5^72^. It is plausible that GOF promotes postnatal cell death and thus reduces the number of neurons at P7. Furthermore, NeuN antibody recognizes both glutamatergic and GABAergic neurons. Thus, whether GABAergic neuronal numbers are altered in GOF brain tissues remains to be further studied in the future.

We postulate that FGFR3 GOF in postmitotic cortical neurons exerts non-cell autonomous influences on RGCs, resulting in the reduction of RGCs and the decrease in cell cycle progression gene expression in GOF mice. When an Ai9 Cre reporter mouse (loxP-flanked STOP cassette preventing transcription of a CAG promoter-driven tdTomato) was crossed to our NEX-Cre line to determine the sites of NEX-Cre mediated recombination, we found that tdTomato^+^ cells did not overlap with PAX6^+^ RGCs at E15.5 (Supplementary Fig. 6), consistent with previous findings^29,74^. GFP derived from the FGFR3 floxed construct also highlighted the upper cortical plate in the VZ/SVZ area as the site of Cre-mediated recombination (Fig. 3). Thus, the data presented here indicate that aberrant FGFR signaling in postmitotic neurons impairs RGC functions. Such cross-talk between postmitotic neurons and RGCs had been shown for cardiotrophin-1^75^, FGF18^76^, Sip1-NTF3^77^, FGF9^78^, and BDNF-BMP7^79^ signaling. Interestingly, we found that *Fgf9* mRNA is significantly upregulated (FDR = 0.0001, Supplementary Dataset 1) in GOF brain tissue. IPA analysis of E15.5 embryonic brain tissue also indicates that BMP signaling pathway (Z score 0.632; Supplementary Dataset 2) is activated in the GOF brain.

### FGFR3 GOF in NEX-lineage neurons leads to malformation of several long-range axonal tracts

Successful axon navigation depends on the competence of the growing tip of the axon to receive and integrate information provided by multiple spatially organized molecular cues arranged along the axon’s trajectory. Utilizing ScaleS clearing brain methodology, we visualized several miss-routed axonal tracts caused by FGFR3 GOF in cortical glutamatergic neurons. These tracts include anterior commissure, cortical striatal projection, and post-commissural fornix (Figs. 7, 8, 9). RNA-seq data from E15.5 brain tissue identified significant dysregulation in axonal guidance signaling pathways, including Slit-Robo, Wnt-Frizzled, and Gli pathway (Fig. 6E). In line with our observations, it has been shown that FGFRs regulate axon guidance cues such as slit and semaphorin3A to control axonal pathfinding^50,53^. Slit-Robo signals regulate the pathfinding of multiple axonal pathways, including corticofugal, thalamocortical, and callosal connection^80–83^. Frizzled-3, one of the members in the Wnt-Frizzled pathway, is required for the development of multiple axon tracts, including anterior commissure, corticofugal axons, thalamocortical axons, and corpus callosum in the mouse CNS^84–87^. When frizzled-3 was removed in the neocortex (Emx1-IRES-Cre), the posterior part of the anterior commissure was completely missing, and axons with aberrant trajectories appeared in the external capsule^84^.

The molecular identity of neurons also determines the neuronal subtype and influence axonal wiring patterns. RNAseq studies revealed that the mRNA expression level of *Ctip2* (also known as *Bcl11b*) was significantly upregulated in the E15.5 FGFR3 GOF brain (Fig. 6B). While CTIP2 immunostaining also revealed increased abundance at the protein level, without changing the total number of CTIP2^+^ neurons (Fig. 2L). We also found that the percentage of SATB2^+^/CTIP2^+^ neurons was significantly increased in FGFR3 GOF cortices (Fig. 2M). It has been shown that SATB2^+^/CTIP2^+^ neurons project to callosal and sub-cerebral targets^88^. Furthermore, ectopic expression of CTIP2 in layer 2/3 neurons is sufficient to alter the axonal targeting of corticocortical projection neurons and cause them to project to subcortical targets^89^. Thus, it is also possible that the axonal tract abnormalities we observed in FGFR3 GOF mice are partially due to increased CTIP2 expression in FGFR3 GOF neurons. *Ctip2* expression is directly regulated by the SATB2 transcriptional factor, which acts as a transcriptional repressor to inhibit CTIP2 promoter activity^90,91^. A recent study further shows that the transcriptional adaptor, Lmo4, inhibits CTIP2 by competing with SATB2 for Hdac1 binding^88^. Interestingly, we found that *Lmo4* mRNA is significantly upregulated (FDR = 0.0476, Supplementary Dataset 1) in FGFR3 GOF brain tissue. Taken together, our data suggest that FGFR3 GOF may increase the *ctip2* mRNA expression level via upregulating Lmo4.

### The impact of FGFR3 GOF varies at different stages of cortical development

*In-situ* hybridization data reveal that FGFR1-3 mRNA levels are abundant in RGCs and IPCs during cortical development but relatively low in the cortical plate, presumably postmitotic neurons^92^ (https://developingmouse.brain-map.org/). Despite the low expression level, the recent RNA-seq data demonstrated that postmitotic neurons also express FGFRs^93,94^. These findings suggest that the downregulation of FGFR3 signaling may be essential for maintaining the balance of neurogenesis and neuronal differentiation during cortical development. Several FGFR3 de novo mutations were identified in thanatophoric dysplasia (TD) patients, including R248C, S249C, K650M, K650E^95–99^. Patients with these mutations have intellectual disability, seizures, and cortical malformations^95,96,100^. In addition to severe skeletal deformities, several neuropathological deficits have been identified in TD patients, such as hippocampal dysplasia, subependymal and subarachnoid neuroglial heterotopia, cytoarchitectural disarray, and thinning of lamination in the cortex^100–104^. The R248C, K650E, and K650M mutations resulting in constitutive activation of FGFR3 tyrosine kinase^97–99^ suggest that FGFR3 hyperfunction causes severe brain malformations. Lin et al*.* provided the first evidence that neural FGFR3^K650E^ expression directly impacts brain development^19^. They generated CNS and cartilage-specific *Fgfr3*^*K644E*^ (corresponding to K650E in humans) mouse models with Nestin-Cre and Col2a1-Cre, respectively, to express *Fgfr3*^*K644E*^ under regulation by its endogenous enhancer/promoter. The larger brain and cortical thickness in the Nestin-Cre *Fgfr3*^*K644E*^ mouse were attributed to increased proliferation and reduced apoptosis of progenitor cells^20,105^. Ectopic neurons accumulating in the dentate gyrus were also observed^19^. Moreover, overexpressing FGF8 ligand to enhance FGFR signaling in a ferret resulted in megalencephaly, polymicrogyria, subependymal heterotopia, and leptomeningeal heterotopia^106,107^. In contrast to previous studies, the GOF mouse generated in this study by overexpressing *FGFR3*^*K650E*^ in NEX-lineage neurons exhibited a severe cortical lamination defect (Figs. 1, 2) and abnormal axonal tracts (Figs. 7, 8, 9, Supplementary Fig. 7–9) as having been observed in TD human patients but have not been reported in previous TD animal models. Our observation that the abnormal cortical lamination and hippocampal patterning is apparent only when FGFR3 is overexpressed in postmitotic neurons, but not postnatally (Supplementary Fig. 3), further underscores the importance of a tightly regulated developmental window. In summary, our data suggest FGFR3 GOF in postmitotic glutamatergic neurons may contribute to brain anatomical changes in TD patients. However, this hypothesis remains to be tested in the animal models where FGFR3 expression is controlled by its endogenous promoter. Nevertheless, the different stages of onset for *FGFR3*^*K650E*^ expression in, for example, progenitor cells vs. postmitotic neurons, clearly impacts brain structures very differently.

## Materials and methods

### Experimental design and animals

The mutant *FGFR3* allele (CAG-flox-stop-flox-*FGFR3*^*K650E*^-IRES-eGFP), NEX-Cre, and NEX-CreERT2 transgenic mice were described previously^29,30,108^. Specifically, a K650E mutation in *FGFR3* was identified in one form of neonatal lethal dwarfism, TD II (OMIM 187,601). This mutation is located in the activation loop of the kinase domain and causes constitutive activation of FGFR3^109,110^. The NEX-Cre allows Cre-mediated recombination to occur in postmitotic cortical and hippocampal glutamatergic neurons from embryonic day 11.5 (E11.5)^29^. The NEX-CreERT2 allows temporal regulation on Cre-mediated recombination with tamoxifen administration^108^. The mixed genetic background from multiple crosses may result in a heterogeneous phenotype. To minimize this effect, littermate controls were used for all the experiments and processed simultaneously with hyperfunction samples. Mice carrying only NEX-Cre or flox *FGFR3*^*K650E*^ allele were used as controls. Heterozygous offspring were also crossed to Ai9 reporter mice (Rosa-CAG-LSL-tdTomato-WPRE; JAX Lab # 007909) to label axon projections of all NEX-lineage principal neurons. Both male and female mice were used in this study. All mice were housed in standard conditions with food and water provided ad libitum and maintained on a 12 h dark/light cycle. All animal breeding and experimental procedures were approved and performed following the guidelines set by Indiana University Bloomington Institutional Animal Care and Use Committee.

### Genotyping

Ear or tail lysates were prepared by immersing tissue in digestion buffer (50 mM KCl, 10 mM Tris–HCl, 0.1% Triton X-100, 0.1 mg/ml proteinase K, pH 9.0), vortexing gently, and then incubating for 3 h at 60 °C to lyse the cells. These lysates were then heated to 94 °C for 10 min to denature the proteinase K (Thermo Scientific, Rockford, IL, USA), and centrifuged at 16,100 *g* for 15 min. The supernatants were used as DNA templates for polymerase chain reactions (PCRs, EconoTaq Plus Green 2X mater mix, Lucigen, Middleton, WI, USA). The genotyping primers were as described previously^29,30,108^.

### Chemicals and antibodies

All reagents and chemicals were purchased from Sigma (St. Louis, MO, USA) unless otherwise stated. D(-)-Sorbitol, glycerol, and urea were purchased from Wako Chemicals USA Inc. (Richmond, VA, USA). Rabbit anti-phosphorylated FGFR substrate 2α (FRS2α, Tyr196, Cat# 3864, RRID: AB_2106222), rabbit anti-phosphorylated extracellular signal-regulated kinase 1/2 (ERK1/2, Thr202/Tyr204, Cat#9101; RRID: AB_331646), and mouse anti-ERK1/2 (Cat# 9107; RRID: AB_2235073) antibodies were purchased from Cell Signaling (Beverly, MA, USA). Rabbit anti-FRS2α (Cat# SC-8318; RRID: AB_2106228) and rabbit anti-CUX1 (Cat# SC-13024; RRID: AB_2261231) antibodies were purchased from Santa Cruz Biotechnology (Santa Cruz, CA, USA). Chicken anti-green fluorescent protein (GFP, Cat#GFP-1020, RRID: AB_10000240) antibody was purchased from Aves Labs (Tigard, Oregon, USA). Rabbit anti S100β (Cat# Z0311; RRID: AB_10013383) antibody was purchased from DAKO (Produktionsvej, Glostrup, Denmark). Guinea pig anti-vesicular glutamate transporter 2 (VGluT2) (Cat#2251-I; RRID: AB_2665454) and Rabbit anti-NeuN (Cat# MAB377; RRID: AB_2298772) was purchased from Millipore (Temecula, CA, USA). Rabbit anti-PAX6 (Cat#901301; RRID: AB_2565003) was purchased from BioLegend (San Diego, CA, USA). Rabbit anti-brain lipid-binding protein (BLBP, Cat# ab32423; RRID: AB_880078), rabbit anti-TBR1 (Cat# ab31940; RRID: AB_2200219), rat anti-phospho Histone H3 (pH3, Cat# ab10543; RRID: AB_2295065), and rabbit anti-TBR2/Eomes (Cat# ab23345; RRID: AB_778267) antibodies were purchased from Abcam (Cambridge, MA, USA). Rat anti-BrdU antibody (Cat# OBT0030G; RRID: AB_609567; which recognized the BrdU derivative IdU) was purchased from Accurate Chemical (Westbury, NY, USA). All Alexa Fluor series conjugated secondary antibodies were purchased from Invitrogen (Grand Island, NY, USA). Secondary antibodies used for western blotting were purchased from LI-COR Biosciences (Lincoln, NE, USA).

### Western blotting

For total protein extraction, brain tissue was lysed and homogenized with a modified radioimmunoprecipitation assay buffer [50 mM of Tris‐base (pH 7.4), 50 mM of NaCl, 1% Triton X‐100, 0.1% sodium dodecyl sulphate, 1 mM of EDTA, 1% Na‐deoxycholate, 1 mM of phenylmethylsulfonyl fluoride, 1 μg/mL of leupeptin, 1 μg/mL of aprotinin, 1 mM of Na_3_VO_4_, and 1 mM of NaF]. The supernatant solution was collected using centrifugation at 12,000×*g* for 15 min at 4 °C. Protein concentration was measured using a Bradford assay (Bio‐Rad, Hercules, CA, USA). Proteins were separated on a 10% SDS–polyacrylamide gel and then transferred to a nitrocellulose membrane (BioRad, Boston, MA, USA). The membrane was incubated with the appropriate primary antibody and then incubated with a species-appropriate secondary antibody. Western blot images were acquired by LI-COR Odyssey scanner and software (LI-COR Biosciences, Lincoln, NE USA) and quantified with NIH ImageJ software.

### Immunostaining

Mouse brain tissues were prepared by intracardiac perfusion with phosphate-buffered saline (PBS) followed by 4% paraformaldehyde (PFA) prepared in PBS. Fixed brains were sectioned into 100 μm thick sections in the coronal plane using a Leica VT-1000 vibrating microtome (Leica Microsystems). Embryonic brain tissues were dissected at specific post-gestation dates, and post-fixed with 4% PFA for 2 h at 4 °C, followed by cryoprotection in 30% sucrose and embedding in optimal cutting temperature compound. Fixed embryonic brains were sectioned into 30 μm thick sections in the coronal plane using a Leica Cryostat CM1850 (Leica Microsystems). Sections were permeabilized with 0.3% Triton X100, then incubated with a blocking solution (3% normal goat serum prepared in PBS with 0.3% Triton X-100) and then incubated overnight with primary antibody prepared in blocking solution. An appropriate secondary antibody conjugated with an Alexa series fluorophore was used to detect the primary antibody. Draq5 (1:10,000 dilution, Cell Signaling) or 4´,6-diamidino-2-phenylindole (DAPI, 5 μg/ml, Invitrogen) were included in the secondary antibody solution to stain nuclei.

### IdU labeling and staining

Time-mated female mice were injected with 100 mg/kg IdU in PBS intraperitoneally at E14.5 or E15.5. Brain sections were prepared as described above in the Immunostaining section. Brain sections were permeabilized, and DNA denatured with 4 N HCl in PBS with 0.1% Triton X-100 for 30 min at 37 °C, followed by neutralizing with 0.1 M sodium borate (pH 8.5) for 15 min at room temperature. Alternatively, sections were incubated with 0.5 unit/µl DNase I (TaKaRa, Kusatsu, Shiga, Japan) for 60 min at 37 °C. After acid treatment or DNase I treatment, sections were subjected to the immunostaining procedure as described above in the Immunostaining section.

### Image acquisition and quantification

The dorsal view of post-fixed brain tissue was imaged by a FMA050 color CCD (AmScope, China). Epi-fluorescent images were taken by a DFC365FX monochrome CCD (Leica). Z-stack confocal images were acquired with a Leica SP8 confocal microscope. Specifically, BLBP^+^ processes were imaged with a 40X/NA 0.95 objective, and the Z-stacks were taken at 0.5 µm intervals, five µm-total thickness was imaged and projected. CUX1^+^, PAX6^+^, TBR1^+^, TBR2^+^, IdU^+^, and pH3^+^ cells were imaged with a 10X/NA0.75 or 20X/NA 0.7 objective, and the Z-stacks were taken at 1 µm intervals. Projection images of 5 µm-thickness were used to count cell numbers. For CUX1^+^, PAX6^+^, TBR1^+^, TBR2^+^, and IdU^+^ cell quantification, the region of interest (ROI) was defined as a rectangle box (with 100 µm width) containing whole cortex (pial to ventricle). For the cell distribution analysis, the entire cortex was further divided into ten bins, and the cell counts were reported as the percentage of the total cell for each bin. For the quantification of PAX6 and pH3 double-positive cells, a rectangle ROI (200 µm × 400 µm) was defined for cell counting. Triple-immunostaining to visualize NeuN^+^, S100β^+^, and IdU^+^ cells were simultaneously imaged with a 10X/NA 0.75 objective with a computational zoom of 0.75 × and taken at 1 µm intervals. Projection images of 5 µm thickness were used to count cell numbers. The length, area, and numbers were quantified by using NIH ImageJ software.

### RNA-seq and pathway analysis

Cortices from E15.5 embryonic brains from control and GOF mice were used for RNA-seq experiments. Total RNA was extracted from brain tissue by RNeasy Mini Kit (Qiagen, Qiagen, Hilden, Germany) and followed by on-column DNase digestion according to the manufacturer’s instruction. RNA sequencing was performed by the Center for Medical Genomics, Indiana University School of Medicine. The concentration and quality of total RNA samples were first assessed using an Agilent 2100 Bioanalyzer. The RNA integrity number of samples was higher than 8.4 for all samples. Total RNA from different biological replicates (n = 5 for control embryos, n = 4 for GOF embryos) was used. Five hundred nanograms of RNA per sample was used to prepare a dual-indexed strand-specific cDNA library using TruSeq Stranded mRNA library Prep Kit (Illumina). The resulting libraries were assessed for their quality and size distribution using Qubind Agilent 2100 Bioanalyzer. One and a half picomoles of pooled libraries were sequenced in the 75 bp single-end configuration on a NextSeeq500 (Illumina) using a NextSeq 500/550 High Output Kit. More than 90% of the sequencing reads reached Q30 (99.9% based call accuracy). The sequencing data were first assessed using FastQC (Babraham Bioinformatics, Cambridge, UK) for quality control. Then all sequenced libraries were mapped to the mouse genome (UCSC mm10) using STAR RNA-seq aligner. Genes with read counts per million (CPM) > 0.2 in more than 3 of the samples were kept. Differential expression analysis was performed using edgeR. False discovery rate (FDR) was computed from *p-*values using the Benjamini–Hochberg procedure. The multiple dimensional scaling (MDS) plot of RNA samples was drawn using plot MDS function in edgeR. The distance represented the leading log-fold-changes between each pair of RNA samples, which is the average (root-mean-square) of the largest absolute log-fold changes between each pair^111^. This visualizes the differences between the expression profiles of different samples in two dimensions. Ingenuity Pathway Analysis (IPA, Qiagen, Germantown, MD) was performed for differentially expressed genes with FDR < 0.05. Enrichment of canonical pathways and disease and bio functions were identified with threshold p < 0.005. The activity of pathways and function was inferred as z-scores. Positive z-score indicated increased activity, while negative z-score indicated inhibited activity.

### Tissue clearing methodology

Brain samples were prepared as described in the Immunostaining section. The fixed brains were sectioned into 1 mm thick sections in the coronal plane using a Leica VT-1000 vibrating microtome (Leica Microsystems). Brain sections were cleared using the Scale protocol^54^, which preserves the red fluorescence signal expressed in this mouse line. Briefly, the brain sections were incubated in ScaleSQ(5) [22.5% d-sorbitol, 9.1 M of urea, and 5% Triton X-100] for 2 h in room temperature, followed by incubation in ScaleS4(D25) [40% d-sorbitol, 10% glycerol, 4 M of Urea, 0.2% Triton X-100, 25% dimethyl sulfoxide] overnight at 4 °C^54^.

### Two-photon image acquisition and data analysis

The 1 mm-thick cleared brain sections were mounted in 100 mm diameter Petri dishes filled with fresh ScaleS4(D25) solution and imaged for 15 ~ 20 h. Images were acquired using a Nikon A1R MP + multi-photon microscope (Nikon Instruments Inc., Melville, NY, USA) equipped with an InSight DeepSee infrared pulsed laser (Spectra-Physics Inc., Santa Clara, USA). The brain section was imaged with a 10X/NA 0.5 objective at 1 µm intervals in the z-axis. Image analysis was performed utilizing the Surface module of Imaris v 9.2 (Bitplane Inc., Zurich, Switzerland). To quantify the volume of axonal projections in the striatal region, a 3-D ROI measuring 800 µm × 800 µm × 700 µm (W × H × D) was selected from the most lateral portion of the striatum visible in the slice containing the anterior part of the hippocampus. The total volume occupied by the cortical glutamatergic axons (tdTomato^+^ axons) was extracted. To account for brain size differences in control and GOF brain, the total volume was further normalized to the area of the corresponding slice. The area of the slice was measured by manually contouring the middle plane of the z-stack image of interest. For the postcommissural fornix analysis, a 3D ROI measuring 2850 µm × 1550 µm × 650 µm (W × H × D) was selected from the portion of the fornix immediately posterior to the anterior commissure. Measurements, including volume, ellipticity prolate, and ellipticity oblate, were extracted. The ellipticity prolate parameter represents the ROI’s similarity to a prolate ellipsoid. The ellipticity oblate parameter represents the ROI’s similarity to an oblate ellipsoid. The ellipticity values range from 0 to 1, being 1 a perfect ellipse (prolate or oblate)^112^. To further investigate the characteristics of postcommissural fornix, four horizontal 2-dimensional (2-D) planes were extracted from the acquired 3-D volume containing the postcommissural fornix, and the maximum diameter of fornix bundle was measured. To illustrate the axonal projections, we prepared a cartoon scheme (Fig. 7C, 9A) utilizing the Scalable Brain Atlas Composer ^113^.

### Data and statistical analysis

Data were analyzed using GraphPad Prism 8.04 software (GraphPad Software, San Diego, CA). In figures, data are expressed as means ± SEM. We employed the unpaired t-test and two-way ANOVA to examine data, as presented in figure legends.

## Supplementary information

Supplementary Information 1.

Supplementary Information 2.

Supplementary Information 3.
