# Supplementary material for: Enhanced FGFR3 activity in postmitotic principal neurons during brain development results in cortical dysplasia and axonal tract abnormality
Source: Sci Rep. 2020 Oct 28;10:18508. doi: 10.1038/s41598-020-75537-0 (PMC7595096; doi:10.1038/s41598-020-75537-0)
Supplement: Supplementary file 1 — Supplementary Information 1. [file 41598_2020_75537_MOESM1_ESM.docx]

**Supplementary Information**

**Title**

Enhanced FGFR3 activity in postmitotic principal neurons during brain development results in cortical dysplasia and axonal tract abnormality

Jui-Yen Huang, Bruna Baumgarten Krebs, Marisha Lynn Miskus, May Lin Russell, Eamonn Patrick Duffy, Jason Michael Graf, Hui-Chen Lu


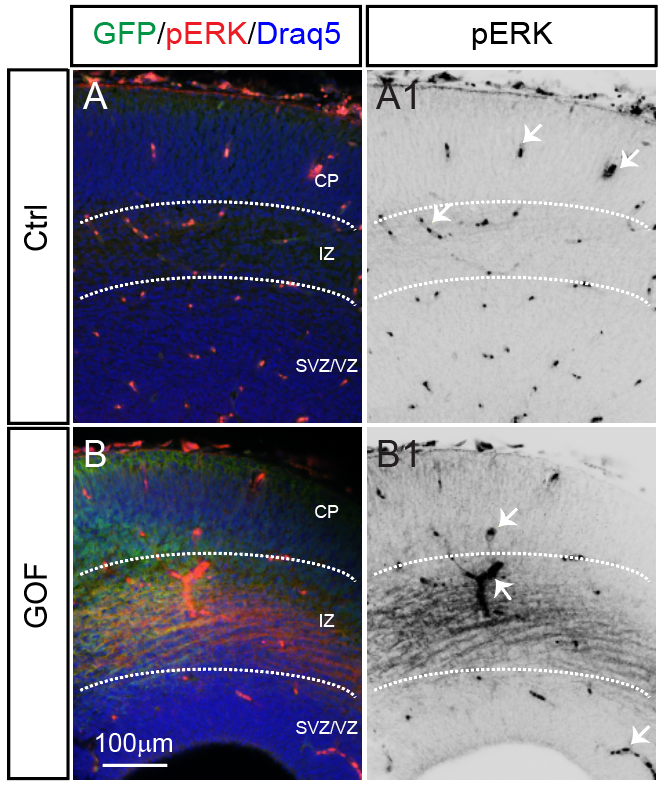


**Supplementary Figure 1. Expressing FGFR3^K650E^ in NEX-lineage neurons results in ERK activation in the E15.5 embryonic brain.** (A, B) GFP and phosphorylated ERK1/2 (Thr202/Tyr204; pERK1/2) staining of coronal sections in E15.5 control (Ctrl) and GOF brains. A1 and B1 show the inverted images of pERK1/2. The immunoreactivity of pERK1/2 located in the intermediate zone (IZ). White arrows indicate the non-specific signal, which usually colocalized with vasculatures. CP, cortical plate; SVZ, subventricular zone; VZ, ventricular zone.


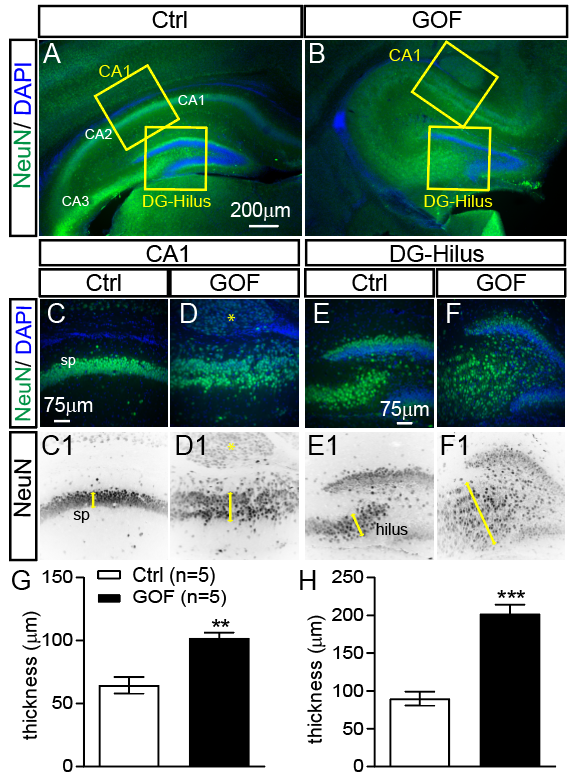


**Supplementary Figure 2. Expressing FGFR3^K650E^ in NEX-lineage neurons results in an aberrant hippocampal structure.** (A, B) NeuN staining of coronal sections showing the hippocampus in P7 control (Ctrl) and GOF brains. (C-F) Higher magnification images in CA1 and dentate gyrus (DG)-hilus (corresponding locations marked with yellow boxes in A and B). C1, D1, E1, and F1 show the inverted images of NeuN. Yellow star indicates a cluster of heterotopic neurons found in GOF brains. (G, H) Summary of the thickness of the NeuN^+^ layer (indicated by the yellow line in C1-F1 (Ctrl, n=5; GOF, n=5) in CA1 (G) and DG-hilus (H). Student’s-t test. **, p < 0.01; ***, p < 0.001.


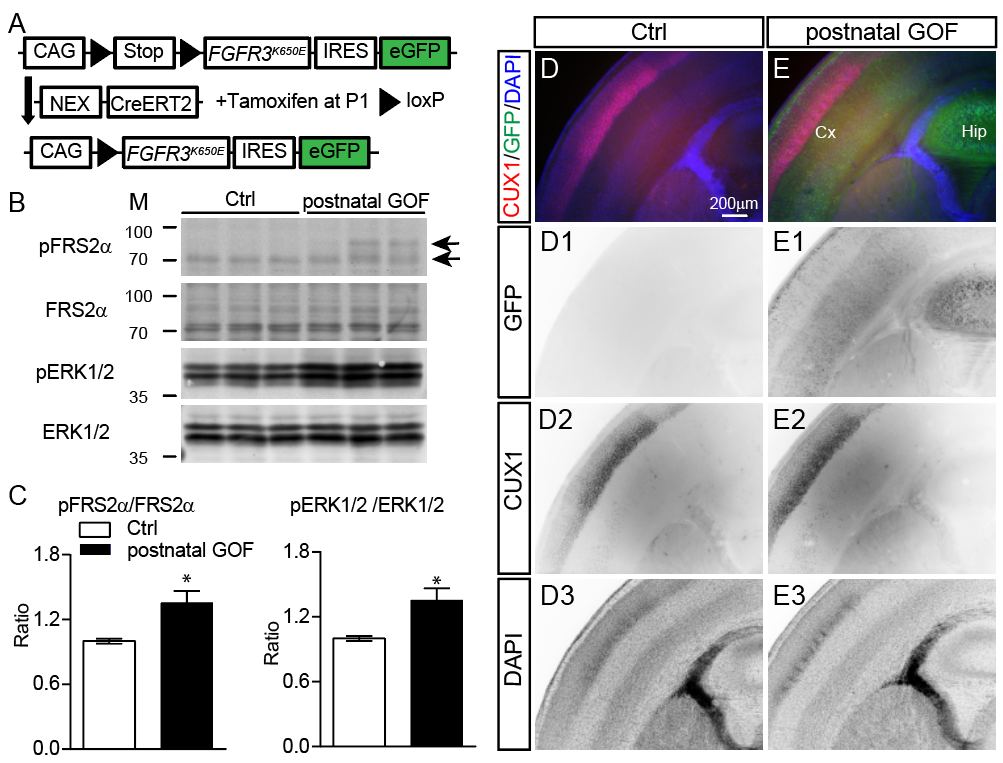


**Supplementary Figure 3. Postnatal FGFR3 GOF did not perturb cortical laminations.** (A) The diagram shows how postnatal-GOF mice were generated. Tamoxifen (100 mg/kg) was injected at postnatal day 1 (P1) to activate CreERT2. The black triangle indicates the loxP site. (B) Western blots show the abundance of pFRS2α, FRS2α, pERK1/2, and ERK1/2 in the S1 cortex of P7 control (Ctrl) and postnatal GOF mice. (C) Summaries for the fold changes of pFRS2α to FRS2α and pERK1/2 to ERK1/2 (Ctrl, n=5; GOF, n=5) in GOF mice. Student’s-t test. * p<0.05. (D-E) GFP and CUX1-staining in the P7 S1 cortex of Ctrl and postnatal GOF brains. D1-D3 and E1-D3 show the inverted images of the indicated channel. Cx, cortex; Hip, hippocampus.


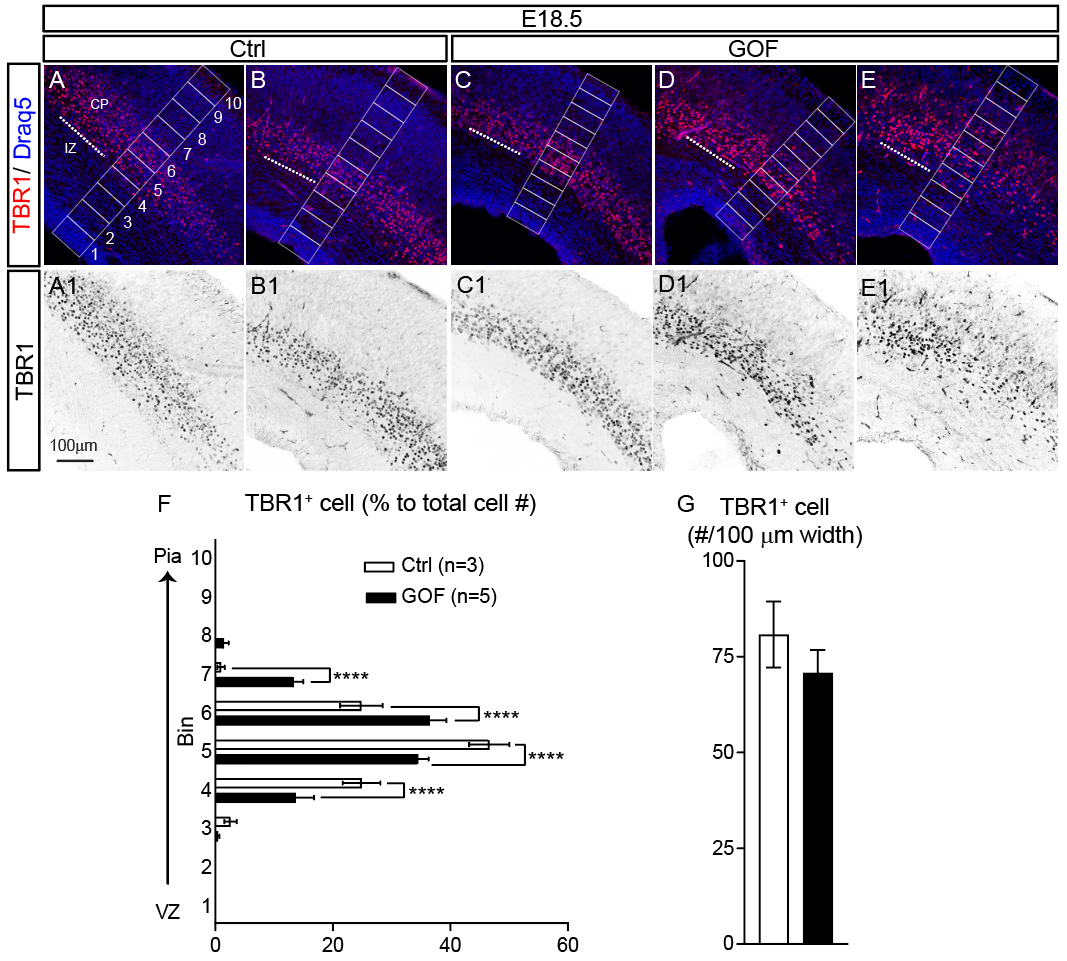


**Supplementary Figure 4. The impact of FGFR3 GOF in postmitotic neurons on layer six neurons. (**A-E) Example images of TBR1 and Draq5 staining with coronal sections prepared from the E18.5 control (Ctrl) and GOF cortex. A1-E1 shows the inverted TBR1 staining images. The white boxes indicated ten bins of same width height for the quantification. The height of bins was adjusted according to the thickness of cortical plate to fit ten bins throughout the cortical plate. 1-10, bin number; CP, cortical plate; IZ, intermediate zone. (F) The distribution of TBR1^+^ cells in E18.5 cortex. Two-Way ANOVA *post hoc* Bonferroni’s multiple-comparisons test. The statistical analysis (*) compared between Ctrl and GOF for corresponding bins. ****, p < 0.0001. (G) Summary for Tbr1^+^ cell numbers within 100 um width of cortical area alone the pial surface with the height across the cortical plate. Student’s-t test.


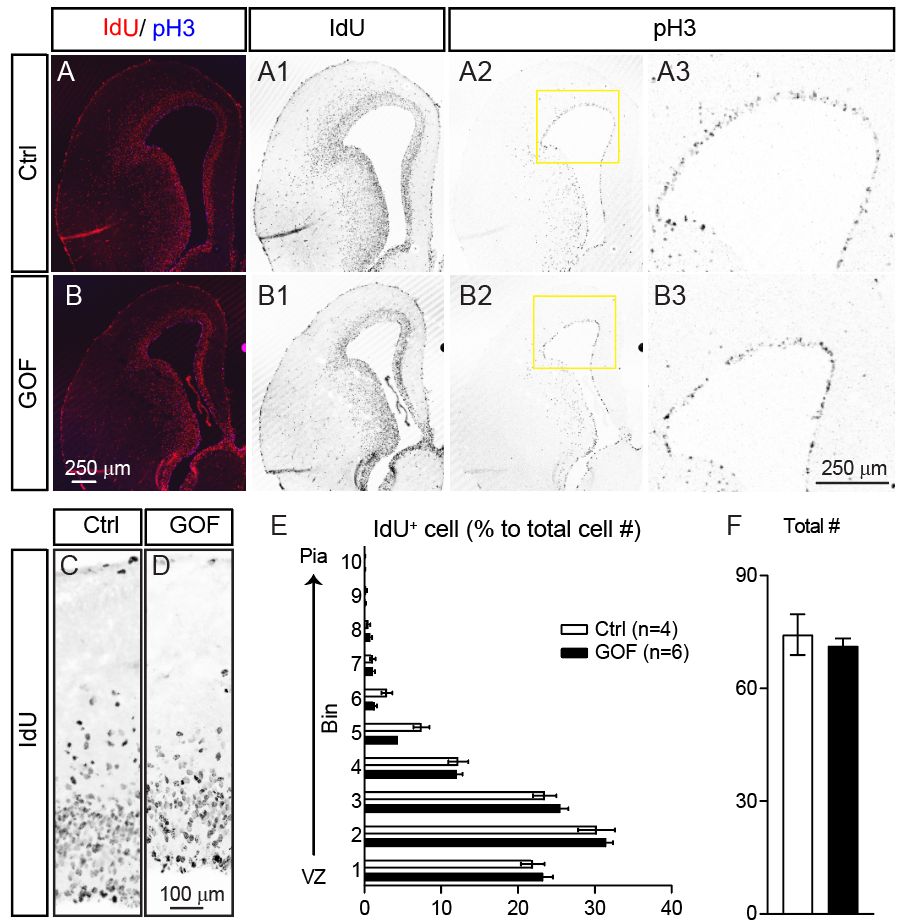


**Supplementary Figure 5. FGFR3 GOF in postmitotic neurons did not alter the IdU^+^ cell number.** IdU was administered to E15.5 pregnant females, and embryonic brains were collected 2 hours after IdU injection. (A, B) Example images of IdU and phospho-histone H3 (pH3) staining. A1, A2, B1, and B2 show the inverted images of the indicated channel. A3 and B3 are the high magnification view of the yellow box in A2 and B2. (C, D) Example images of IdU staining. (E) The distribution of IdU^+^ cells. Two-Way ANOVA *post hoc* Bonferroni’s multiple-comparisons test. (F) Summary of the total cell number of IdU^+^ cells. Student’s-t test.


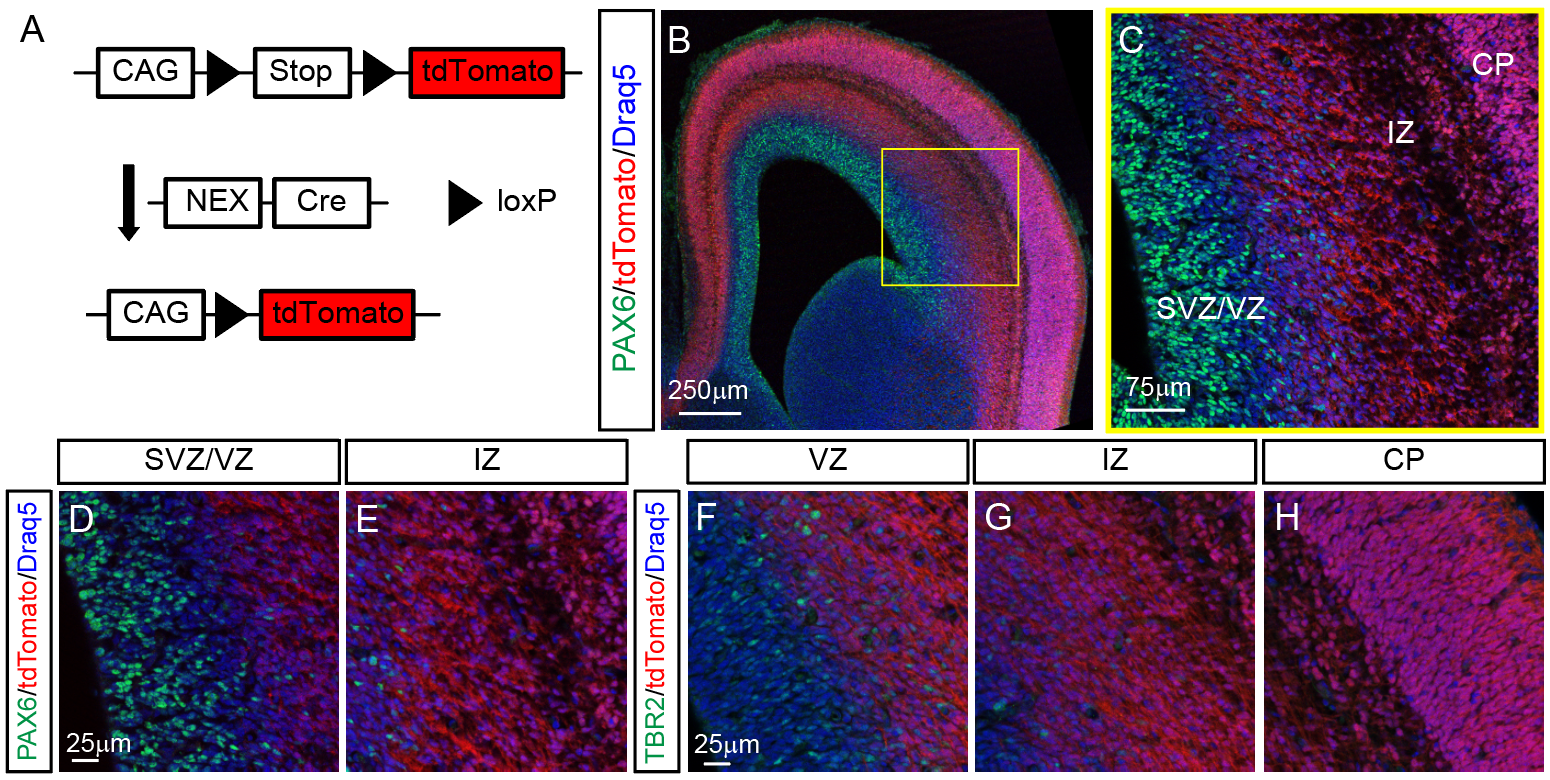


**Supplementary Figure 6. tdTomato reporter mice demonstrate that the majority of Cre mediated recombination occurs in postmitotic neurons.** (A) The diagram shows how NEX-Cre; TdTomato mice were generated. The black triangle indicates the loxP site. (B, C) Images show tdTomato and PAX6 double staining in E15.5 brains. C is the high magnification view of yellow box in B. (D, E) Images for PAX6 and tdTomato double staining in sub-ventricular zone (SVZ)/ventricular zone (VZ) and intermediate zone (IZ). (F-H) TBR2 and tdTomato staining in VZ, IZ, and cortical plate (CP).


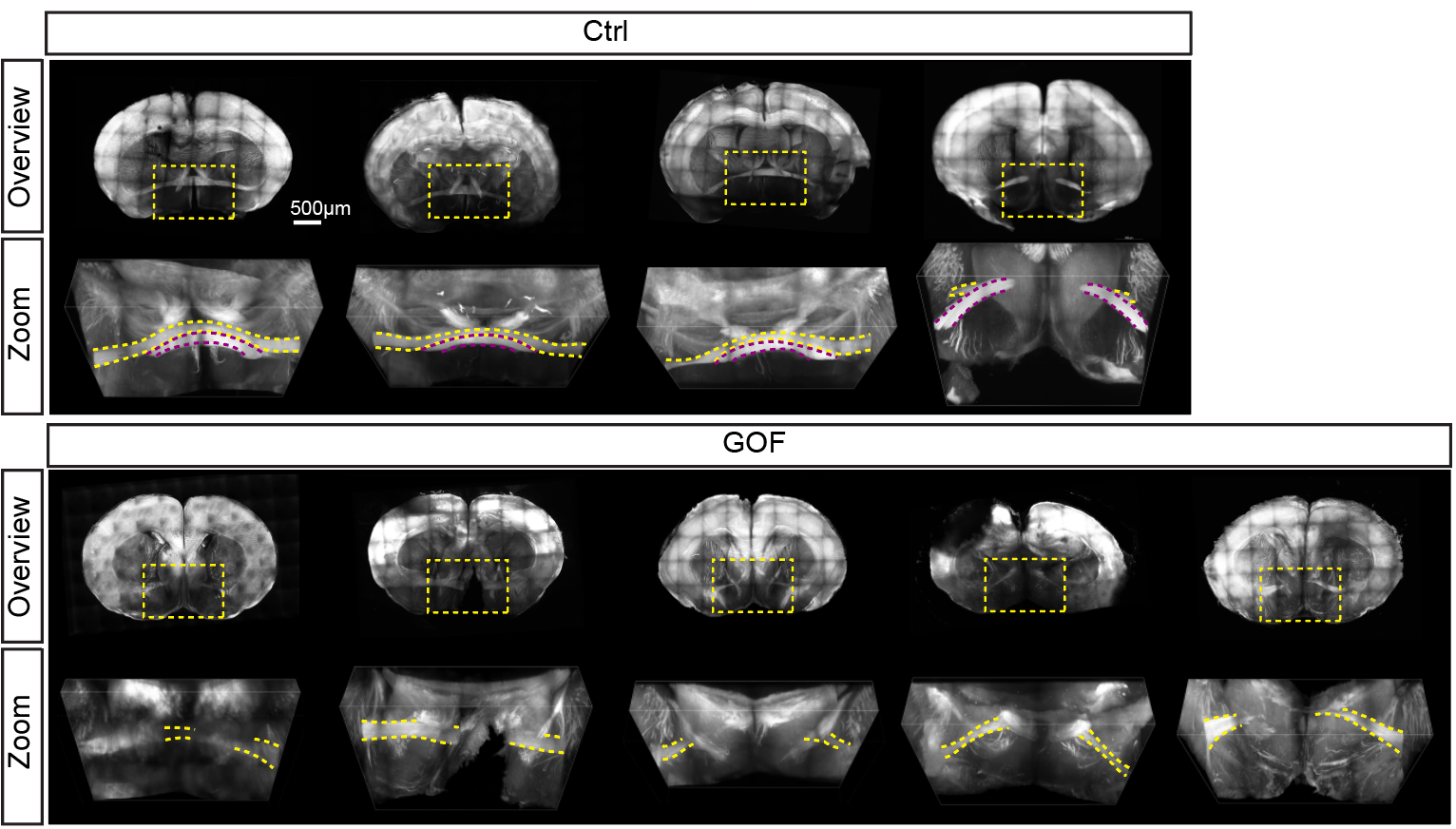


**Supplementary Figure 7. GOF mice have no olfactory limb of the anterior commissure**. More images from different animals to show that the olfactory limb of the anterior commissure is missing in GOF mice.


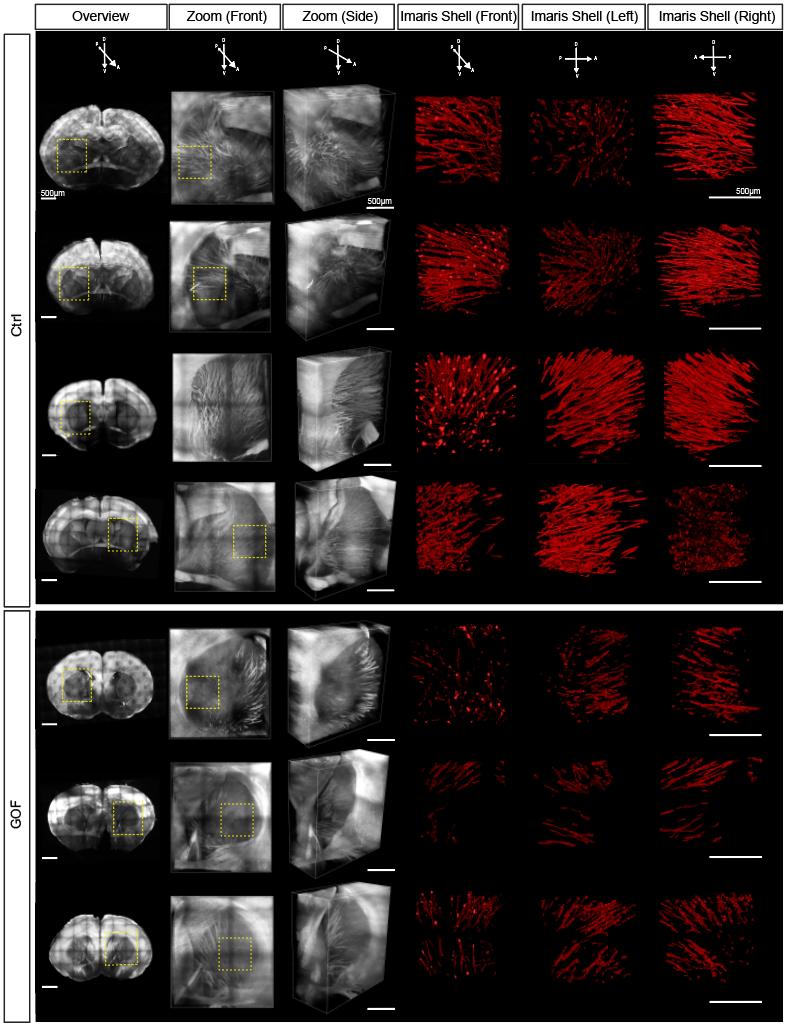
**Supplementary Figure 8. FGFR3 GOF mice have reduced numbers of axonal projections in the striatum.** More images from different animals. D, dorsal; V, ventral, A, anterior; P, posterior.


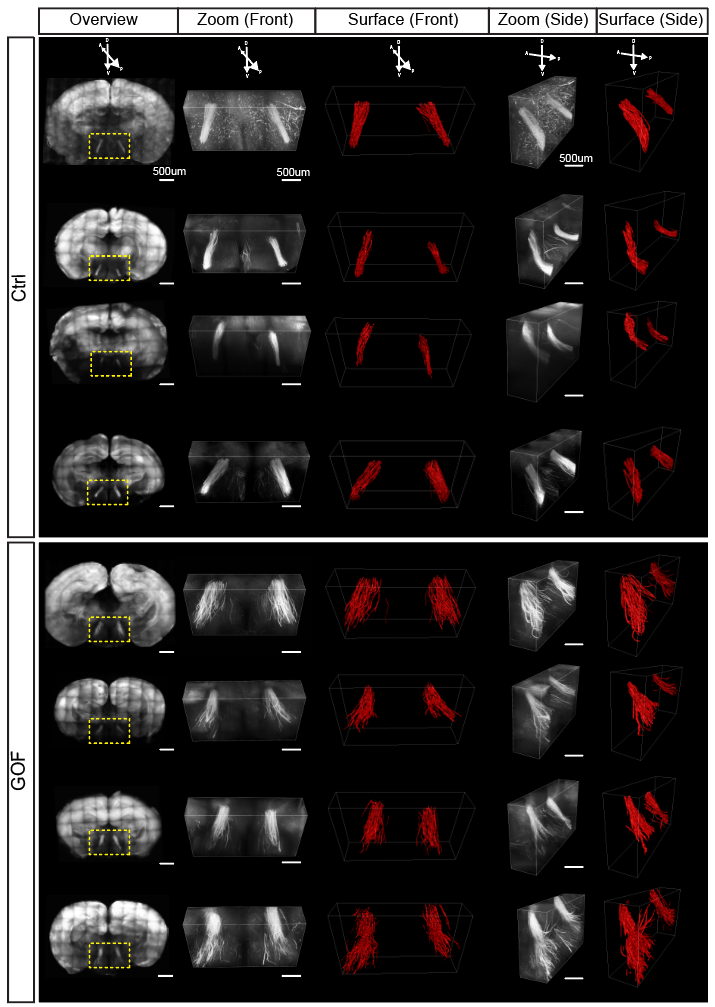
**Supplementary Figure 9. FGFR3 GOF disrupts axonal fasciculation of postcommissural fornix.** More images from different animals. D, dorsal; V, ventral, A, anterior; P, posterior.


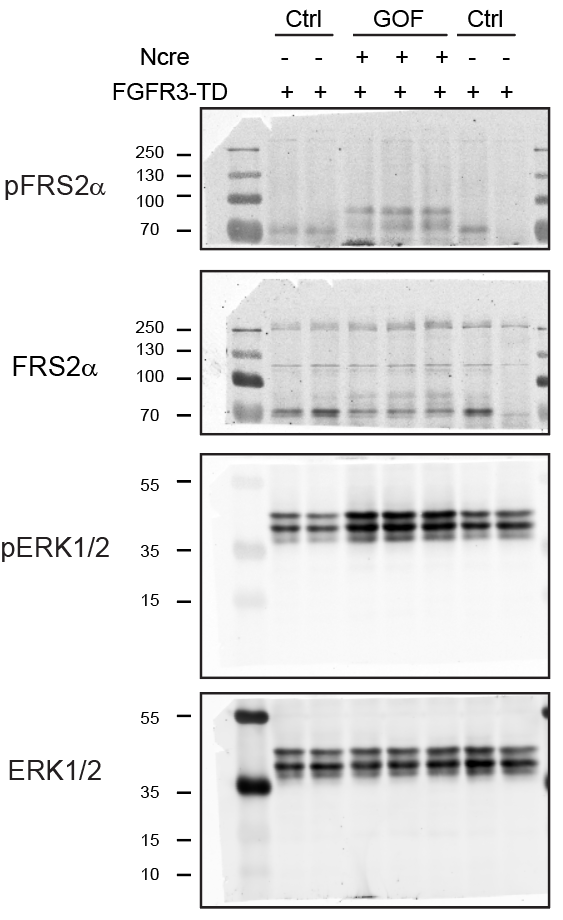


**Supplementary Figure 10.** Full-length blots of Figure1.

**Supplementary Dataset 1.** The list of differentially expressed gene.

**Supplementary Dataset 2.** The canonical pathway analysis.
